# Supplementary material for: A direct coupled electrochemical system for capture and conversion of CO2 from oceanwater
Source: Nat Commun. 2020 Sep 4;11:4412. doi: 10.1038/s41467-020-18232-y (PMC7474062; doi:10.1038/s41467-020-18232-y)
Supplement: Supplementary file 1 — Supplementary Information [file 41467_2020_18232_MOESM1_ESM.pdf]

Supplementary Information

A Direct Coupled Electrochemical System for Capture and Conversion of CO_2 from Oceanwater

Ibadillah A. Digdaya[1], Ian Sullivan[1], Meng Lin[2]*, Lihao Han[1], Wen-Hui Cheng[3], Harry Atwater[3]* and Chengxiang Xiang[1]*

[1]*Joint Center for Artificial Photosynthesis, and Division of Chemistry and Chemical Engineering, California Institute of Technology, Pasadena, California 91125, United States.*

[2]*Department of Mechanical and Energy Engineering, Southern University of Science and Technology, Shenzhen 518055, China.*

[3]*Joint Center for Artificial Photosynthesis, and Department of Applied Physics and Materials Science, California Institute of Technology, Pasadena, California 91125, United States.*

E-Mail: linm@sustech.edu.cn, haa@caltech.edu, cxx@caltech.edu

CO_2 capture from oceanwater cells

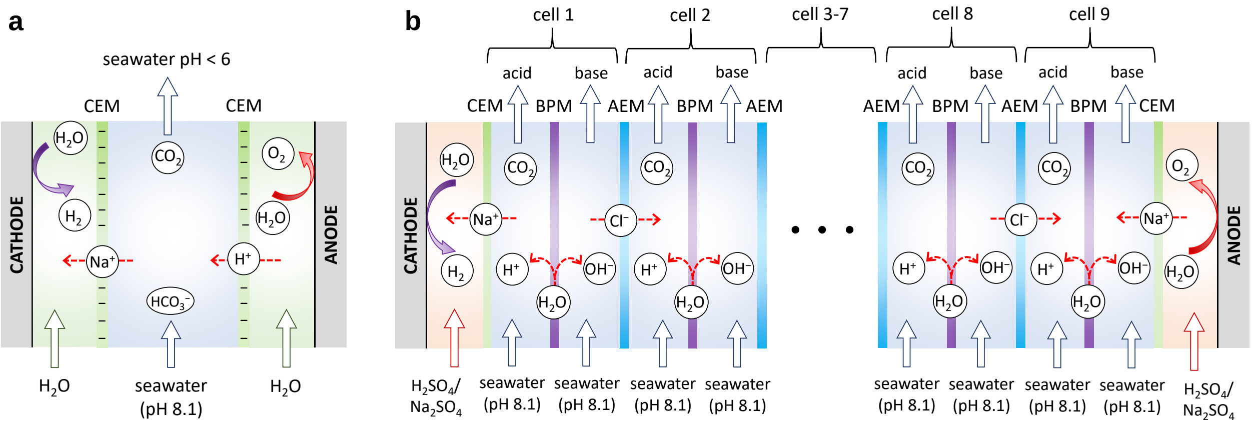

Supplementary Fig. 1 | (a) Schematic of electrolytic cation exchange module (E-CEM) based on continuous electrodeionization (CEDI) principles (adopted from Ref. 1).[1] The E-CEM consists of an anode compartment, a seawater compartment, and a cathode compartment. Each electrode compartment is separated from the seawater with a cation exchange membrane (CEM). The feed for the unit is the natural Key West seawater that is pre-filtered using spin-down filters. The electrode solution is the fresh water with a conductivity of 200 µS cm^{-1} processed from reverse osmosis (RO) of the natural Key West seawater. Water oxidation occurs at the anode and the CEM allows the transport of protons (H^+) from the anode compartment to the seawater compartment. In a simultaneous step, the majority cations (Na^+) in the seawater compartment are transported to the cathode compartment. (b) Schematic of the 9-cell bipolar membrane electrodialysis (BPMED) unit connected in series (adopted from Ref. 2).[2]. Each cell consists of a basified compartment, a bipolar membrane (BPM), an acidified compartment, and an anion exchange membrane (AEM). At each end of the membrane stack, a cation exchange membrane (CEM) is used to separate the membrane stack from the electrode compartment. The seawater is composed of 35.95 g of Instant Ocean® sea salt in 1 liter of deionized water. Each volume element of seawater passes through the system exactly once. The electric field facilitates water dissociation at the BPM and allows H^+ to transport to the acidified compartment and OH^- to transport to the basified compartment. The electrolyte solutions are 0.1 M H_2SO_4/0.25 M Na_2SO_4 and the water splitting reactions take place at the electrodes.

Supplementary Note 1

Multiphysics modeling of the bipolar membrane electrodialysis

A multi-physics 1-dimensional model is developed for the bipolar membrane (BPM) with five calculations domains, cation exchange layer (CEL), junction layer (JL), anion exchange layer (AEL), and two boundary layers (BL) (Supplementary Fig. 2). The length for each domain is listed in Supplementary Table 1.

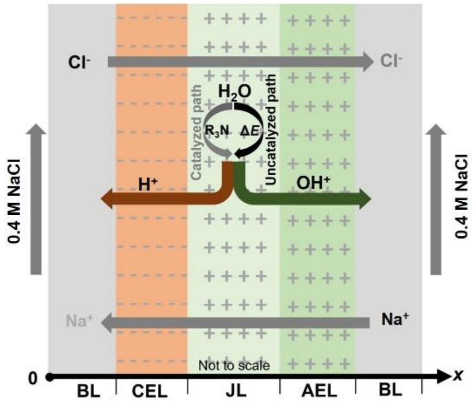

Supplementary Fig. 2 | Schematic of the numerical model of the BPM (not to scale) including 1D domains of boundary layers (BL), cation exchange layer (CEL), junction layer (JL), and anion exchange layer (AEL).

Supplementary Table 1 | Calculation domain lengths.

Domains	Length
BL	50 um
AEL	75 um
CEL	75 um
JL	10 nm

The model considers the electrostatics, species transport, and chemical reactions. The electrostatics and the electrical field of the calculation domains are captured by Poisson equations:

$$\frac{d(\varepsilon E)}{dx} = F(c_{\mathrm{H}^+} - c_{\mathrm{OH}^-} + c_{\mathrm{Na}^+} - c_{\mathrm{Cl}^-} + c_{\mathrm{NR}_2H^+} \pm c_{\mathrm{fix}}) \tag{1}$$

$$-\frac{d\phi}{dx} = E \tag{2}$$

where ε is the dielectric permittivity, F is the Faraday constant, c_i is the concentration of each spices, c_{fix} is the fix charge density in BPM (1 M for both AEL(+) and CEL(−)), E is electrical field, ϕ is the electrostatic potential.

The species transport phenomena are modeled using Nernst-Planck formulation:

$$N_i = -\frac{D_i c_i}{RT}\frac{d\mu_i}{dx} \tag{3}$$

$$\frac{dN_i}{dx} = R_i \tag{4}$$

where N_i species flux, D_i is the diffusion coefficient of each species, R_i is the species mass source term resulting from ionic reactions. D_i in BL are the intrinsic diffusion coefficient in bulk electrolyte which are listed in Supplementary Table 2. While in other domains, D_i are modified using ruggeman approximation with a factor of $f_{el}^{1.5}$ (f_{el} is the fraction of liquid electrolyte in membrane). In this study the liquid electrolyte fraction is assumed to be 17.5%.

Supplementary Table 2 | Intrinsic diffusion coefficient for each species.

Ion	Diffusion coefficient
H^+	9.3×10^{-5} cm^2 s^{-1}
OH^-	5.3×10^{-5} cm^2 s^{-1}
Na^+	1.33×10^{-5} cm^2 s^{-1}
Cl^-	2.03×10^{-5} cm^2 s^{-1}

Three homogeneous chemical reactions are considered in this model including water dissociation reaction (non-catalytical reaction) as well as protonation and deprotonation of weak-based group (catalytical reactions). The electrical potential gradient based water dissociation reaction (Supplementary Eq. (5), R1) is accounted for all domains:

$$H_2O \xleftrightarrow{k_1,k_{-1}} H^+ + OH^- \quad (5)$$

Both k_1 and k_{-1} have strong dependence on the electric field, hence, water dissociation only takes place at the BPM interface where large electric field exists due to the uncompensated fixed charges in the anion exchange layer (AEL) and cation exchange layer (CEL). For the catalytic pathway, the water dissociation is assisted by the protonation and deprotonation of weak base group (-R_3N) from the AEL. The homogeneous chemical reactions (Supplementary Eq. (6) and (7), R2 and R3) and for the catalytic pathway are only considered at the junction layer at the BPM interface.

$$R_3N \xleftrightarrow{k_2,k_{-2}} R_3NH^+ + OH^- \quad (6)$$

$$R_3NH^+ \xleftrightarrow{k_3,k_{-3}} R_3N + H^+ \quad (7)$$

Note that k_1, k_{-1} and k_2, k_{-2} have the same electric field dependence, while k_3 and k_{-3} are independent of the electric field. The intrinsic reaction rate constants without electrical field for both forward and backward reactions of all three reactions are listed in Supplementary Table 3. The operation temperature of the whole membrane is assumed to be 25 °C and hence the temperature effect is neglected in this model.

Supplementary Table 3 | Intrinsic reaction rates without electrical field.

Reactions	Forward constant (k_0 , m^3 mol^{-1}s^{-1})	Backward constant (k_{0-}, m^3 mol^{-1}s^{-1})
R1	3.6734×10^{-10}	1.1143×10^{8}
R2	24.445	2.1312×10^{7}
R3	1.8020×10^{-5}	2.1533×10^{7}

The reaction rates of R1 and R2 are dependent also on the electric field. The enhancement effect of electric field on R1 and R2 is considered by multiplying an enhancement factor (f_{enhance}) to the intrinsic reaction rate constant. The forward ($f_{\text{enhance},\, f}$) and backward ($f_{\text{enhance},\, b}$) reactions enhancement factors for R1 and R2 is calculated based on empirical correlations:[3]

$$f_{\text{enhance},\, f} = \left(\sum_{m=0}^{\infty} \frac{(2b)^m}{m!\,(m+1)!}\right)\cosh(\tau b)(\cosh(\tau))^b \tag{8}$$

$$f_{\text{enhance},\, b} = 1 + \frac{1-\exp\left(-\frac{1}{\alpha}\right)}{2}\left(\alpha^2 b + 4.97\alpha\frac{\sinh(0.0835\alpha b)}{\cosh(0.0835\alpha b)^2}\right) \tag{9}$$

Where α = 0.81 is dimensionless length based on Bjerrum length, $b = 4.8E$ is the dimensionless electric field, and $\tau = -0.128\ln(\cosh(0.235\alpha)) + 5.72\alpha^2$ is a fitting parameter.

The model is solved numerically using finite element method via Newton method in COMSOL Multiphysics. The relative tolerance is set to be 10^{-4} with a mesh number of 6500. The mesh independent study is conducted to minimize the effect of mesh on the results.

Supplementary Note 2

In oceanwater, carbon dioxide is stored as dissolved inorganic carbon (DIC), and exists in three main forms: as dissolved aqueous carbon dioxide, CO_2, as bicarbonate (HCO_3^-), and as carbonate (CO_3^{2-}). The carbonate species are related to the following equilibria:

$$CO_2 + H_2O \rightleftharpoons HCO_3^- + H^+ \rightleftharpoons CO_3^{2-} + 2H^+ \quad (10)$$

For the description of carbonate system in oceanwater, stoichiometric equilibrium constants are used:

$$K_1 = \frac{[HCO_3^-][H^+]}{[CO_2]} \quad (11)$$

$$K_2 = \frac{[CO_3^{2-}][H^+]}{[HCO_3^-]} \quad (12)$$

where K_1 ($10^{-5.86}$, $pK_1 = 5.86$)[4] is the first dissociation constant and K_2 ($10^{-8.92}$, $pK_2 = 8.92$)[4] is the second dissociation constant of carbonic acid.

The sum of the dissolved forms CO_2, CO_3^{2-} and HCO_3^- is the DIC:

$$\text{DIC} = [HCO_3^-] + [CO_3^{2-}] + [CO_2] \quad (13)$$

The charge balance of the carbonate system in oceanwater can be expressed by total alkalinity (TA):

$$\text{TA} = [HCO_3^-] + 2[CO_3^{2-}] + [B(OH)_4^-] + [OH^-] - [H^+] + \text{minor components} \quad (14)$$

The $[CO_3^{2-}]$ is counted twice because it has double negative charge. For synthetic oceanwater, boron and other minor components can be neglected,[5] and for typical oceanwater at pH 8.1, the contribution of water alkalinity ($[OH^-] - [H^+]$) is very small. The charge balance of the simplified oceanwater at normal conditions can then be expressed by carbonate alkalinity (CA):

$$\text{CA} = [HCO_3^-] + 2[CO_3^{2-}] \quad (15)$$

The input oceanwater solutions used in the experiments were synthetic oceanwater prepared by adding 35.95 g of Instant Ocean® sea salt per liter of deionized water.

Supplementary Table 4 | The concentration of major constituents of the synthetic oceanwater prepared by adding 35.95 g per liter deionized water as analyzed by ion chromatography (Dionex™) and the concentration major constituents of natural oceanwater.

Constituents	Instant Ocean® synthetic oceanwater (mM)	Natural oceanwater (mM)[6]
Na^+	416.643	480.61
Mg^{2+}	56.678	54.13
Ca^{2+}	11.766	10.52
K^+	9.096	10.46
NH_4^+	0.122	n.a.
Li^+	0.09	n.a.
Sr^{2+}	n.a.	0.092
Cl^-	505.333	559.39
SO_4^{2-}	14.789	28.935
HCO_3^-	2.69*	1.891
CO_3^{2-}	0.408*	0.189
CO_2	0.0155*	0.0133
Br^-	1.185	0.863
NO_3^-	0.111	n.a.
CH_3COO^-	0.108	n.a.
F^-	0.026	0.070

* Determined from carbonate alkalinity

Alkalinity titration of synthetic oceanwater

In order to quantify the DIC of the synthetic oceanwater, alkalinity titration was performed on 100 ml oceanwater sample using 0.01 M HCl titrant solution. The titrant was dropped slowly into the sample and the change of pH was monitored and plotted as a function of the added titrant volume (Supplementary Fig. 3).

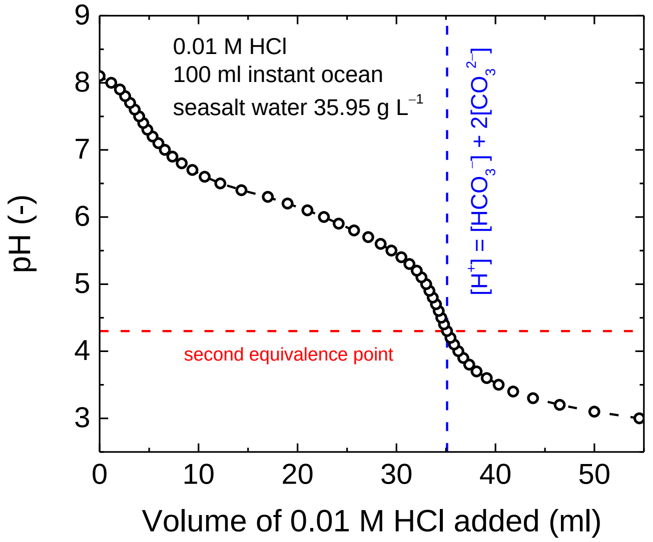

Supplementary Fig. 3 | Alkalinity titration curve of synthetic oceanwater (35.95 g of Instant Ocean® sea salt per liter deionized water).

At the second equivalence point, or at pH 4.3 the concentration of H^+ is equal to the sum of $[HCO_3^-]$ and $2[CO_3^{2-}]$.[5] For the simplified oceanwater, the number of moles of H^+ that is added to neutralize the anions and weak acid is approximately equals to CA:

$$\text{CA} = [\text{H}^+]_{\text{added}} = [\text{HCO}_3^-] + 2[\text{CO}_3^{2-}] \quad (16)$$

To reach pH 4.3, 35 ml of 0.01 M HCl was added to 100 ml of oceanwater sample, and the number of moles per liter of added H^+ can be calculated as follows:

$$[\text{H}^+]_{\text{added}} = 35 \text{ ml of titrant} \times \frac{1 \text{ L}}{1000 \text{ ml}} \times \frac{0.01 \text{ mol H}^+}{1 \text{ L}} \times \frac{1}{0.1 \text{ L oceanwater sample}} \quad (17)$$

From Supplementary Eq. (17), the $[H^+]$ added to bring the pH of the oceanwater sample down to 4.3 is 3.5 mM, which is equal to the sum of $[HCO_3^-]$ and $2[CO_3^{2-}]$.

Using Supplementary Eq. (11)and (12), the $[HCO_3^-]$ was 2.696 mM, the $[CO_3^{2-}]$ was 0.408 mM and the $[CO_2]$ was 0.016 mM. Using Supplementary Eq. (13), the DIC was calculated to be 3.12 mM.

The concentration of $[HCO_3^-]$, $[CO_3^{2-}]$ and $[CO_2]$ as a function of $[H^+]$ or pH is expressed by the following relationships:

$$[\text{HCO}_3^-] = \text{DIC} \Big/ \left(1 + \frac{[\text{H}^+]}{K_1} + \frac{K_2}{[\text{H}^+]}\right) \quad (18)$$

$$[\text{CO}_3^{2-}] = \text{DIC} \Big/ \left(1 + \frac{[\text{H}^+]}{K_2} + \frac{[\text{H}^+]^2}{K_1 K_2}\right) \quad (19)$$

$$[\text{CO}_2] = \text{DIC} \Big/ \left(1 + \frac{K_1}{[\text{H}^+]} + \frac{K_1 K_2}{[\text{H}^+]^2}\right) \quad (20)$$

The concentration of $[HCO_3^-]$, $[CO_3^{2-}]$ and $[CO_2]$ in the synthetic oceanwater as a function of pH is illustrated by Bjerrum plot in Supplementary Fig. 4.

At temperature (T) of 25 °C and salinity (S) of 35, the logarithmic form of the water dissociation constant (pK_W) is 13.22,[4] and is given by:

$$\text{p}K_\text{W} = \text{pH} + \text{pOH} \quad (21)$$

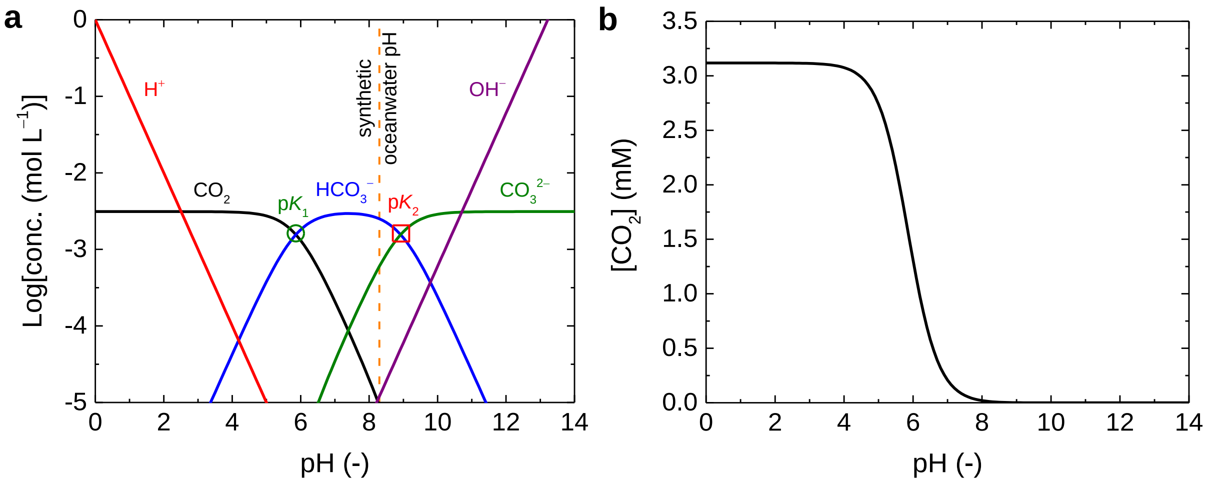

Supplementary Fig. 4 | (a) Bjerrum plot of log concentrations (conc.) of HCO_3^-, CO_3^{2-}, CO_2, H^+, and OH^- as a function of pH of the synthetic oceanwater; DIC = 3.12 mM, S = 35, T = 25 °C. The circle and square indicate pK_1 and pK_2, respectively. The value of pK_W is 13.22. (b) CO_2 concentration as a function of pH in the synthetic oceanwater.

Simulated partial current densities across the BPM

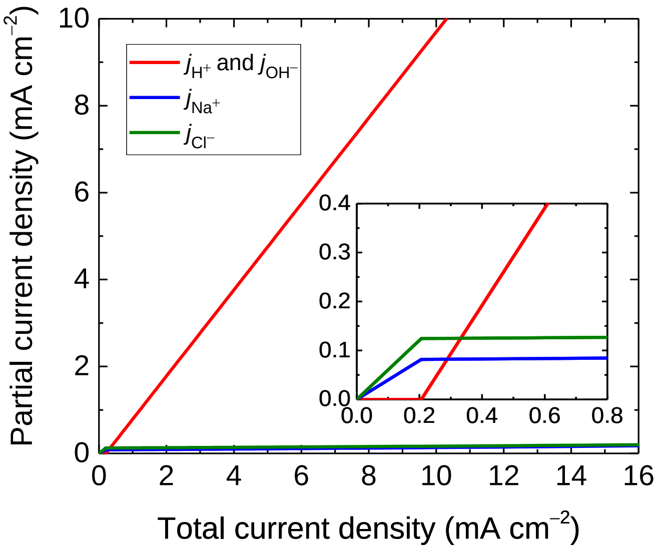

Supplementary Fig. 5 | Partial current density carried by H^+ (j_{H^+}), OH^- (j_{OH^-}), Na^+ (j_{Na^+}) and Cl^- (j_{Cl^-}) across the BPM as a function of the total current density (j_{total}).

pH *vs.* current calculation

The generation rate of H^+ (G_{H^+}, in mol s^{-1}) and OH^- (G_{OH^-}) due to electrical current can be expressed by:

$$G_{H^+} = \frac{I_{H^+}}{nF} \tag{22}$$

$$G_{OH^-} = \frac{I_{OH^-}}{nF} \tag{23}$$

$$G_{H^+} = G_{OH^-} \tag{24}$$

where I_{H^+} is the absolute partial current carried by H^+ (in A), I_{OH^-} is the absolute partial current carried by OH^-, n is the number of participating electron per generation of one H^+ and one OH^- in a single stack cell ($n = 1$), and F is the Faraday constant (96485.3329 s A mol^{-1}).

The number of H^+ that is added (in mol L^{-1}, M) in the acidified stream is given by the rate of generated H^+ divided by the rate of the oceanwater flow (Q_{OW}, in L s^{-1}) in the acidified compartment of the BPMED cell.

$$[H^+]_{added} = \frac{G_{H^+}}{Q_{OW}} \tag{25}$$

The number of H^+ that is required to acidify oceanwater to a certain pH can be estimated by keeping track the charge balance of the carbonate system. Any H^+ that is introduced to the oceanwater will be consumed by HCO_3^- and CO_3^{2-} until a new equilibrium is reached, and the oceanwater pH is determined by the equilibrium concentration of H^+. Therefore, the number of added H^+ in the oceanwater is related with the change in concentrations of HCO_3^-, CO_3^{2-}, OH^- and H^+ from the initial values (*i.e.*, $pH_{initial} = 8.1$), as given by:

$$[H^+]_{added} = \Delta[HCO_3^-] + \Delta 2\left[CO_3^{2-}\right] + \Delta[OH^-] - \Delta[H^+] \tag{26}$$

The concentrations of HCO_3^-, CO_3^{2-}, OH^- as a function of H^+ or pH can be derived from Supplementary Eq. (18), (19), (20) and (21), as also shown in the Bjerrum plot (Supplementary Fig. 4a).

Using the relationship between j_{H^+} and j_{total} in Supplementary Fig. 5, and using Supplementary Eq. (22), (25) and (26), the pH of the oceanwater can be plotted as a function of the electrical current. By normalizing the absolute current with the oceanwater input flow rate, the oceanwater pH is independent of the membrane and electrode active area, as shown in Supplementary Fig. 6.

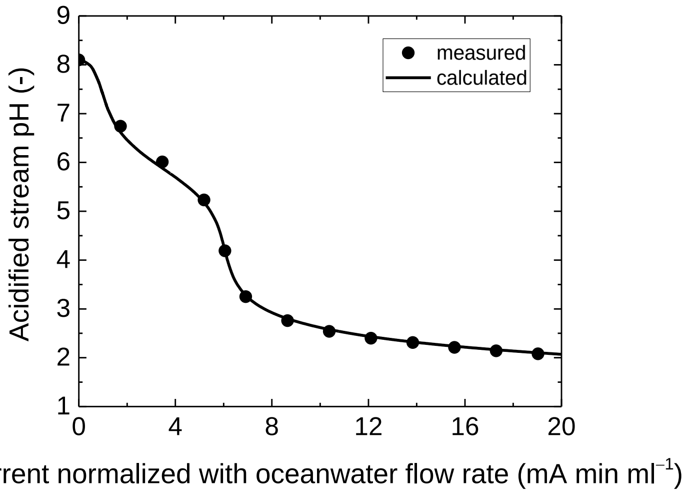

Supplementary Fig. 6 | The experimental and calculated acidified stream pH as a function of electrical current normalized with oceanwater flow rate.

Supplementary Note 3

Electrodialysis energy consumption and CO_2 output flow rate calculations

The electrochemical energy consumption ($E_{electrochemical}$, in kWh kg^{-1} CO_2) used for the contour plot was estimated by the following relationship:

$$E_{\text{electrochemical}} = \frac{VI}{[\text{CO}_2]_{\text{pH}} Q_{\text{OW}} M_{\text{CO}_2}} \tag{27}$$

where V, the cell voltage determined from the experimental voltage–current density characteristic of the cell containing 0.4 M K_3/K_4-Fe(CN)$_6$ redox couple electrode solution, I is the absolute current, $[CO_2]_{pH}$ is the concentration of CO_2 (in mol L^{-1}) at the given applied current density or pH according to Supplementary Eq. (20), (22), (25) and (26), and M_{CO_2} is the molar mass of CO_2 (0.044 kg mol^{-1}).

The calculation steps to determine the electrochemical energy consumption are outlined in the Supplementary Fig. 7.

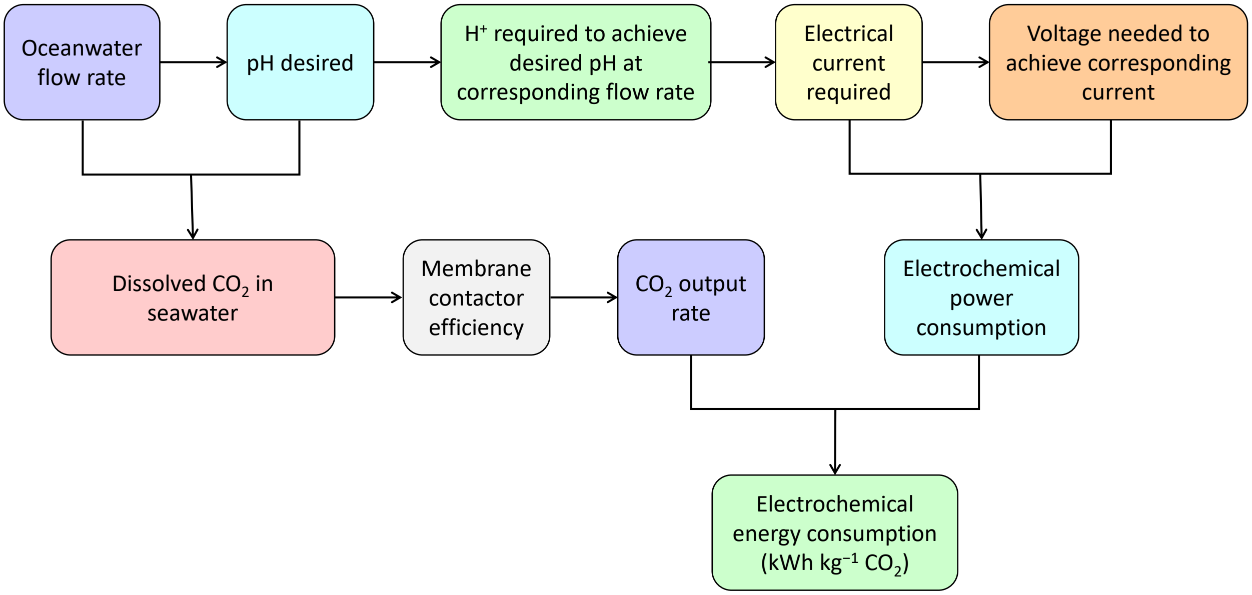

Supplementary Fig. 7 | Flowchart outlining calculation steps of electrochemical energy consumption.

The generation rate of H^+ required to acidify oceanwater to a desired pH at the given oceanwater flow rate was determined by Supplementary Eq.(25) and (26). The electrical current needed to generate H^+ at the desired rate was calculated based on the partial current density and the total current density relation in Supplementary Fig. 5 and using Supplementary Eq. (22). The electrochemical power consumption was determined from the required total current density and the voltage from the experimental voltage–current density curve. The electrochemical energy consumption was approximated using Supplementary Eq. (27).

The ideal CO_2 output rate ($Q_{CO_2,\text{ ideal}}$) depends on the concentration of dissolved CO_2 in oceanwater at the given pH and the oceanwater flow rate, as given in the following relationship:

$$Q_{CO_2,\text{ ideal}} = [CO_2]_{pH} Q_{OW} \frac{RT}{P} \tag{28}$$

where R is the gas constant (0.082057 L atm K^{-1} mol^{-1}), T is the temperature (in K) and P is the pressure (1 atm).

The experimental electrochemical energy consumption ($E_{\text{electrochemical, exp}}$, in kWh kg^{-1} CO_2) was determined from the following equation:

$$E_{\text{electrochemical, exp}} = \frac{V_{exp} I_{exp}}{Q_{CO_2,\text{ measured}} M_{CO_2} \frac{P}{RT}} \tag{29}$$

where V_{exp} is the observed cell voltage, I_{exp} is the experimental applied absolute current, and $Q_{CO_2,\text{ measured}}$ is the measured output of CO_2 flow rate.

The CO_2 capture efficiency ($\eta_{capture}$) was calculated by dividing the measured rate of the CO_2 output with the rate of DIC (Q_{DIC}) at the given oceanwater flow rate:

$$\eta_{capture} = \frac{Q_{CO_2,\text{ measured}}}{Q_{DIC}} = \frac{Q_{CO_2,\text{ measured}}}{[DIC] Q_{OW} \frac{RT}{P}} \tag{30}$$

The membrane contactor efficiency (η_{MC}) was calculated as follows:

$$\eta_{MC} = \frac{Q_{CO_2,\text{ measured}}}{Q_{CO_2,\text{ ideal}}} \tag{31}$$

BPMED cell voltage at 3.3 mA cm^{-2}

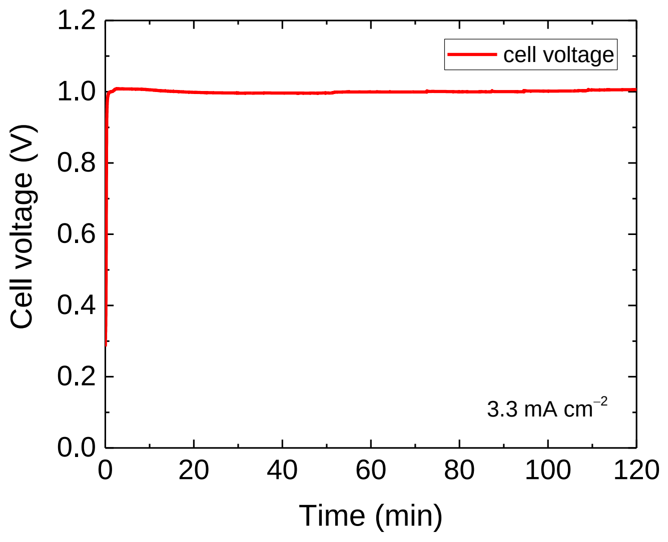

Supplementary Fig. 8 | Cell voltage of the BPM electrodialysis unit recorded during CO_2 capture experiment at an operating current density of 3.3 mA cm^{-2}.

CO_2 capture performance with vacuum stripping at the BPMED cell outlet only

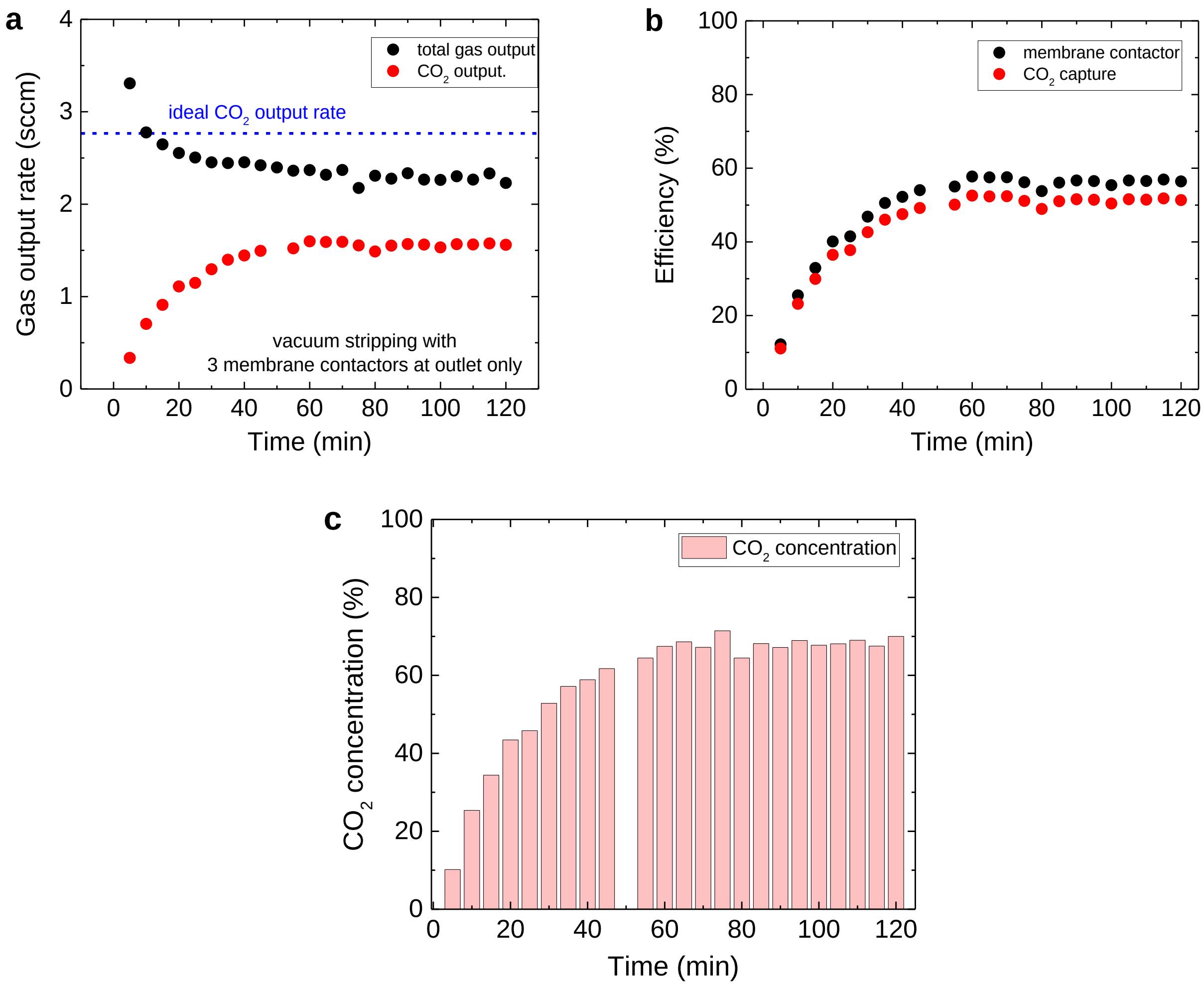

Supplementary Fig. 9 | (a) Total gas output rate and the CO_2 output rate, (b) membrane contactor and CO_2 capture efficiency, and (c) concentration of CO_2 output of the system that use 3 membrane contactors at the outlet of the BPMED cell only and no vacuum stripping stage prior to the BPMED, measured at an operating current density of 3.5 mA cm^{-2} and oceanwater flow rate of 40 ml min^{-1} as a function of time. The blue dash line in panel (a) indicates the ideal rate of CO_2 output at these operating conditions (at pH 4.7 and an oceanwater flow rate of 40 ml min^{-1}).

CO_2 capture with vacuum stripping through one membrane contactor at the inlet and outlet of the BPMED cell

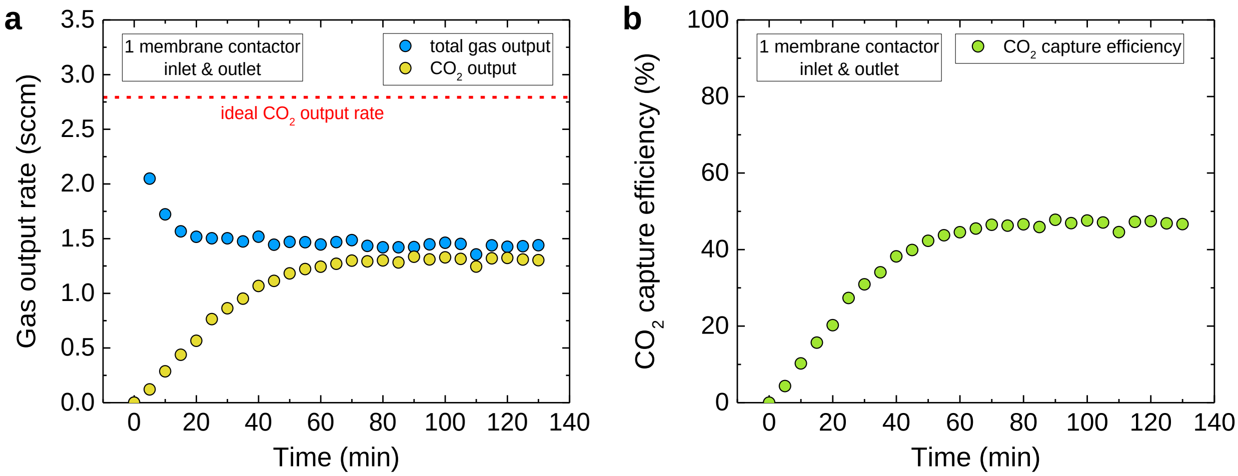

Supplementary Fig. 10 | (a) The total gas output rate and the CO_2 output rate, and (b) the CO_2 capture efficiency of the system that used 1 membrane contactor at the inlet and at the outlet of the BPMED cell at an oceanwater flow rate of 37 ml min^{-1} acidified to pH 3.7 as a function of time. At this pH, all DIC had converted to dissolved CO_2. The red dash line in panel (a) indicates the ideal rate of CO_2 output at these operating conditions.

CO_2 capture with vacuum stripping through two membrane contactors at the inlet and outlet of the BPMED cell

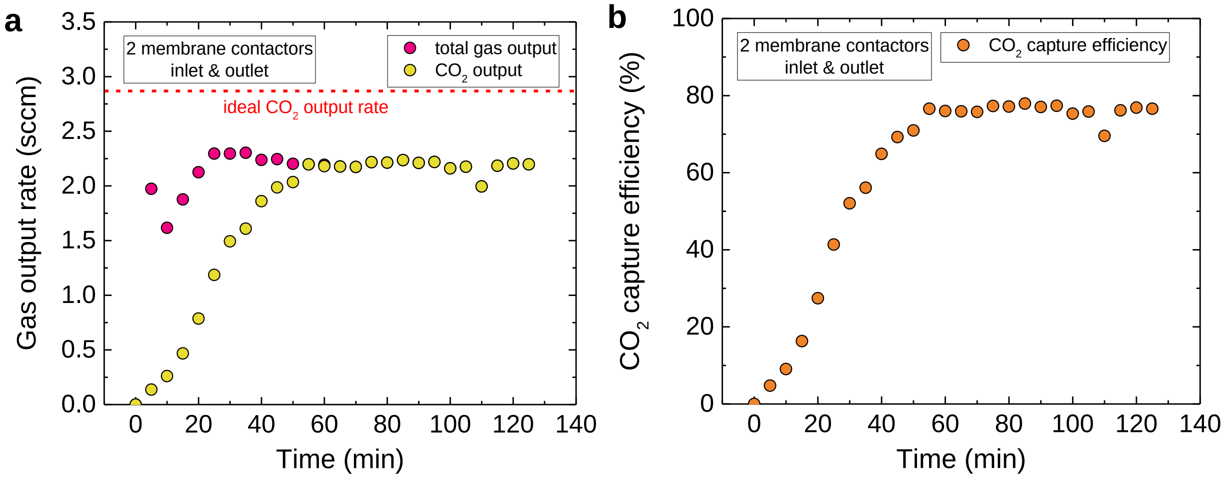

Supplementary Fig. 11 | (a) The total gas output rate and the CO_2 output rate, and (b) the CO_2 capture efficiency of the system that used 2 membrane contactors at the inlet and at the outlet of the BPMED cell at an oceanwater flow rate of 38 ml min^{-1} acidified to pH 3.7 as a function of time. At this pH, all DIC had converted to dissolved CO_2. The red dash line in panel (a) indicates the ideal rate of CO_2 output at these operating conditions.

CO_2 capture with vacuum stripping through three membrane contactors at the inlet and outlet of the BPMED cell

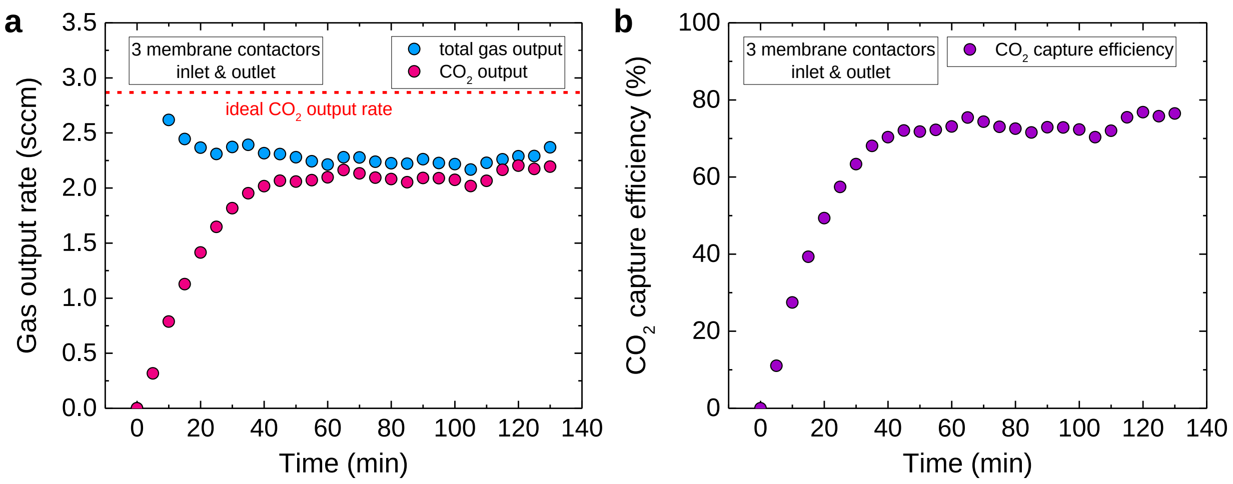

Supplementary Fig. 12 | (a) The total gas output rate and the CO_2 output rate, and (b) the CO_2 capture efficiency of the system that used 3 membrane contactors at the inlet and at the outlet of the BPMED cell with oceanwater flowing at a rate of 37 ml min^{-1} and acidified to pH 3.7 as a function of time. At this pH, all DIC had converted to dissolved CO_2. The red dash line in panel (a) indicates the ideal rate of CO_2 output at these operating conditions.

Supplementary Note 4

At an oceanwater flow rate of 37 ml min^{-1}, a current density of 3.3 mA cm^{-2}, the pH of the acidified stream was 4.7, and the calculated $[CO_2] = 2.92 \times 10^{-3}$ M, $[HCO_3^-] = 2.02 \times 10^{-4}$ M, $[CO_3^{2-}] = 1.22 \times 10^{-8}$ M, $[H^+] = 2 \times 10^{-5}$ M and $[OH^-] = 3.02 \times 10^{-9}$ M.

At 76% membrane contactor efficiency, 76% of dissolved $[CO_2]$ at pH 4.7 was removed from the acidified stream, and the remaining $[CO_2] = 7.01 \times 10^{-4}$ M. The removal of CO_2 from the oceanwater allowed the carbonate species to re-equilibrate and prompted the pH to adjust according to the new equilibrium condition. Using Supplementary Eq. (11), the new equilibrium concentration of H^+ was 4.8×10^{-6} M, the resulting pH was 5.32 and the new DIC concentration was 9.02×10^{-4} M.

The new equilibrium concentrations of the CO_2, HCO_3^-, CO_3^{2-}, H^+ as a function of pH can be calculated using Supplementary Eq. (18), (19) and (20). The equilibrium concentration of OH^- was calculated using Supplementary Eq. (21) with a pK_W value of 13.22 for oceanwater.[4]

During the experiment, the decarbonized acidified oceanwater was supplied to the base compartment where it was basified. The $[OH^-]$ that is introduced to the basified stream due to water dissociation at the BPM under applied electrical current is related to the change in concentrations of CO_2, HCO_3^-, H^+ and OH^- from the pre-basified values (*i.e.*, $pH_{\text{pre-basified}} = 5.32$), as given by the carbonate acidity, as follows:

$$[OH^-]_{\text{added}} = 2\Delta[CO_2] + \Delta[HCO_3{}^-] + \Delta[H^+] - \Delta[OH^-] \tag{32}$$

At a current density of 3.3 mA cm^{-2} and an oceanwater flow rate of 37 ml min^{-1}, the number of $[OH^-]$ that was generated in the base compartment was equal to the number of $[H^+]$ that was generated in the acid compartment, and was calculated to be 3.3×10^{-3} M. The new equilibrium concentrations of the carbonate species, H^+ and OH^- of the basified stream should therefore be $[CO_2] = 6.3 \times 10^{-10}$ M, $[HCO_3^-] = 2.52 \times 10^{-5}$ M, $[CO_3^{2-}] = 8.77 \times 10^{-4}$ M, $[H^+] = 3.45 \times 10^{-11}$ M and $[OH^-] = 1.75 \times 10^{-3}$ M, and the resulting pH of the basified stream should be 10.46.

However, measurements of oceanwater pH of the basified stream indicated a value of 8.5, close to the pre-acidified condition ($pH_{\text{pre-acidified}} = 8.1$). This discrepancy was attributed to the presence of non-negligible amounts of Mg^{2+} and Ca^{2+} in the synthetic oceanwater, which preferentially reacted with OH^- and formed white precipitates of $Mg(OH)_2$ and $Ca(OH)_2$.

In order to support this hypothesis, separate experiments were performed using simplified oceanwater that contained only 0.5 M NaCl and 0.25 mM $NaHCO_3$, and synthetic oceanwater that was prepared by adding 35.95 g Instant Ocean® sea salt per liter deionized water, containing approximately 0.42 M NaCl, 3.12 mM DIC, 56.7 mM Mg^{2+} and 11.8 mM Ca^{2+} (as listed in Supplementary Table 4). During each experiment, the fresh oceanwater (either containing NaCl/$NaHCO_3$ or Instant Ocean® sea salt) was supplied to both the acid and base compartments of the BPMED. Figure 13a shows that in the absence of the Mg^{2+} and Ca^{2+}, the pH of the basified stream reached pH > 10 when the acidified stream pH < 5, in excellent agreement with the calculated values. In contrast, in the presence of Mg^{2+} and Ca^{2+} as in the Instant Ocean® synthetic oceanwater, the pH of the basified stream was well below the theoretical values (Supplementary Fig. 13).

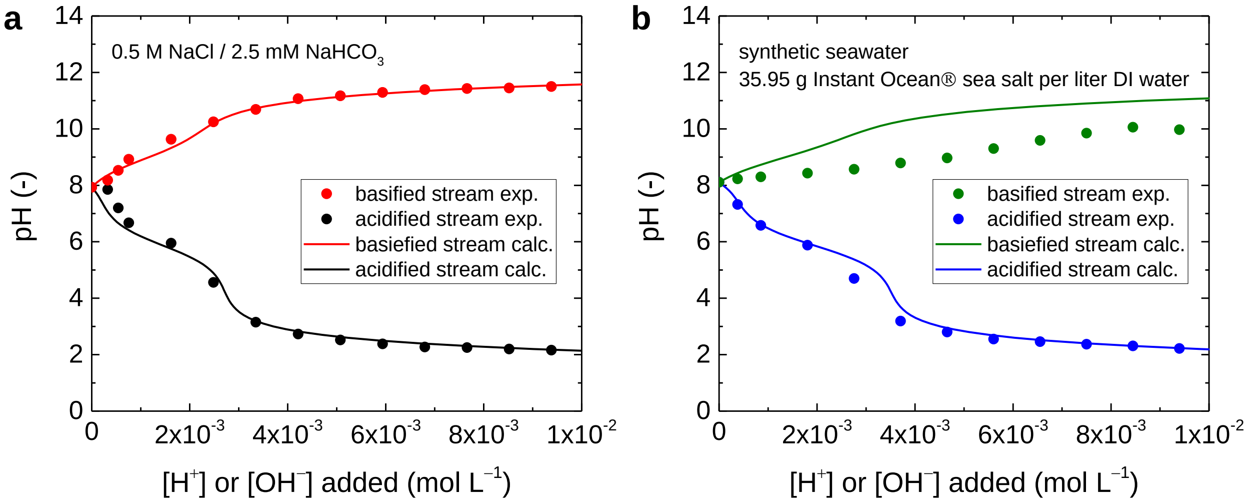

Supplementary Fig. 13 | The experimentally measured (exp.) and calculated (calc.) pH as a function $[H^+]$ added for the acidified stream, and as a function of $[OH^-]$ added for the basified stream from the BPMED using (a) simplified oceanwater containing only 0.5 NaCl and 2.5 mM $NaHCO_3$, and (b) Instant Ocean® synthetic oceanwater containing approximately 3.12 mM DIC, 56.7 mM Mg^{2+} and 11.8 mM Ca^{2+}. The calculated pH values in the panel (b) do not take into account the preferential reaction of OH^- with Mg^{2+} and Ca^{2+}. The $[H^+]$ and $[OH^-]$ added were determined by dividing the calculated generation rate of H^+ (in the acid compartment) and OH^- (in the base compartment) as a result of applied current with the oceanwater flow rate (Supplementary Eq. (22) and (23), respectively). A pK_W value of 13.69 was used to calculate the pH of the solution containing 0.5 M NaCl,[7] and a pK_W value of 13.22 was used to calculate the pH of the Instant Ocean® synthetic oceanwater.[4] Values of pK_1 = 5.86 and pK_2 = 8.92 for carbonate system were assumed to be the same for both cases.

Supplementary Note 5

The cost of BPM (in \$ kg^{-1} CO_2) can be calculated using the following relationship:

$$\text{BPM cost}\left(\frac{\$}{\text{kg}}\right)=\frac{\text{BPM cost}\left(\frac{\$}{\text{cm}^2}\right)}{\frac{j(\text{A/cm}^2)}{nF(\text{A s/mol})}\eta_{\text{MC}}(\%)\frac{[\text{CO}_2]}{[\text{H}^+]}3600\left(\frac{\text{s}}{\text{hour}}\right)M_{\text{CO}_2}\left(\frac{\text{kg}}{\text{mol}}\right)\text{lifetime}} \quad (33)$$

where the BPM cost per area unit is \$0.06 cm^{-2} as quoted from industry partners, j is the current density, n is the number of participating electron (1), F is the Faraday constant (96485.3 A s mol^{-1}), η_{MC} is the membrane contactor efficiency, M_{CO_2} is the molar mass of CO_2, the BPM lifetime is 3 years,[8] and $[CO_2]/[H^+]$ = 2.09 mM/2.39 mM is the ratio between the CO_2 produced and the H^+ introduced in the acidification process of the BPMED for natural oceanwater with a DIC concentration of 2.2 mM, and is the highest at pH 4.6 (**Supplementary Fig. 14**).

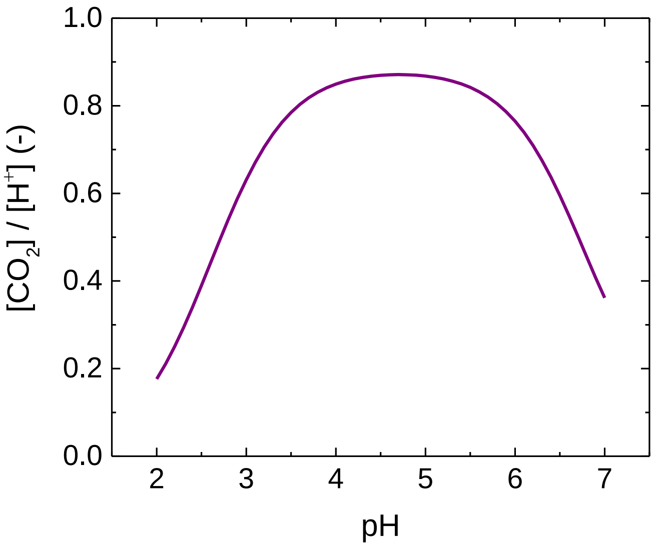

Supplementary Fig. 14 | The ratio between CO_2 produced and H^+ added as a function pH, determined using 2.2 mM DIC as in natural oceanwater.

The cost of CEM (in \$ kg^{-1} CO_2) was assumed to be 1/3 the cost of BPM.

The cost of electrochemical energy (\$ kg^{-1} CO_2) can be determined by the following relationship:

$$\text{Energy cost}\left(\frac{\$}{\text{kg}}\right)=\frac{VI\ (\text{kW})\ \text{elecricity price}\left(\frac{\$}{\text{kWh}}\right)}{\frac{I(\text{A})}{nF(\text{A s/mol})}\eta_{\text{MC}}(\%)\frac{[\text{CO}_2]}{[\text{H}^+]}\ 3600\left(\frac{\text{s}}{\text{hour}}\right)M_{\text{CO}_2}\left(\frac{\text{kg}}{\text{mol}}\right)} \quad (34)$$

The electricity price was assumed to be \$0.06 kWh^{-1}. The voltage is a function of the current density, and was estimated using the simulation (Supplementary Fig. 15)

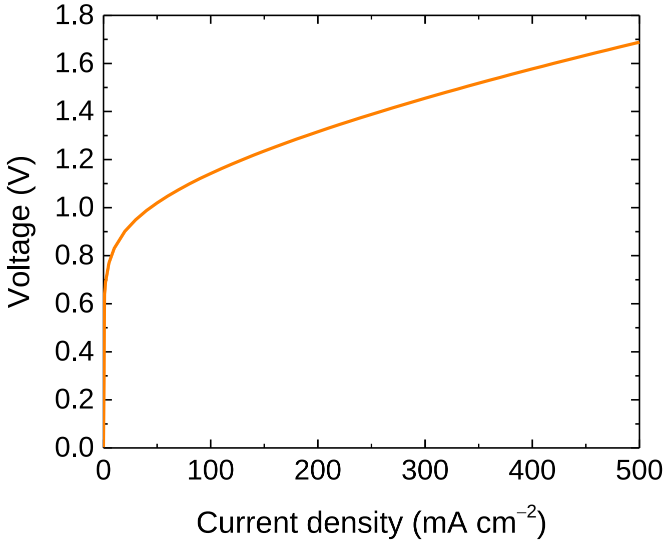

Supplementary Fig. 15 | Simulated voltage-current density characteristics of the water dissociation reaction at the BPM of the electrodialysis unit, taking into account the resistance in the oceanwater.

The trade-offs between the electrochemical energy cost and the membrane cost in the overall capture cost of CO_2 in the BPMED system at different current densities is shown in Supplementary Fig. 16.

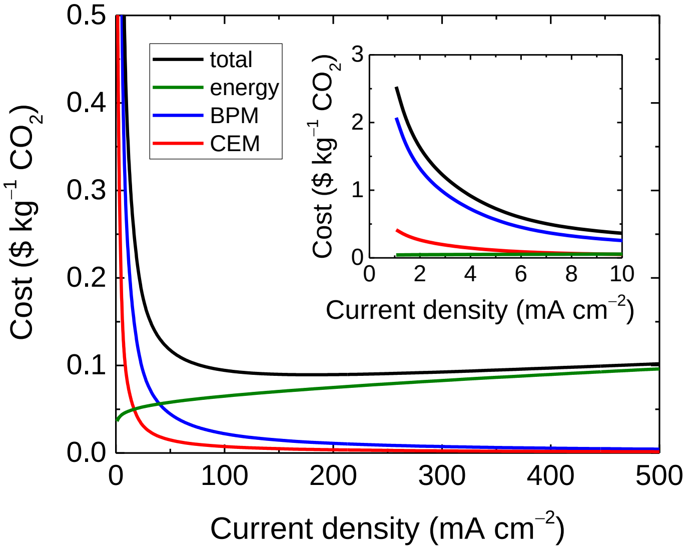

Supplementary Fig. 16 | The cost of CEM, BPM and electrochemical energy (in \$ kg^{-1} CO_2) as a function of applied current density. CEM costs reflect the major capital cost of the electrodialysis unit and the electrochemical energy reflects the major operating cost. The inset highlights the cost at low current densities operation.

Electrochemical conversion of CO_2 from oceanwater

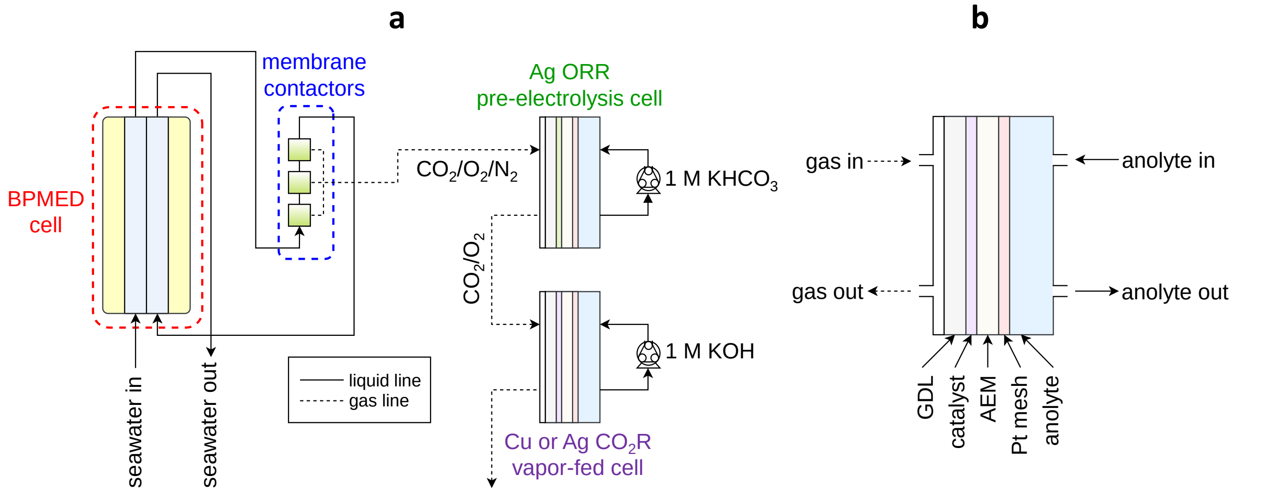

Supplementary Fig. 17 | (a) Process flow of electrochemical conversion of CO_2 captured from oceanwater. The CO_2 output from the BPMED cell was fed through tandem electrochemical cells. The first O_2 reduction reaction (ORR) pre-electrolysis cell was used to eliminate any O_2 residue from flowing into the CO_2 reduction (CO_2R) cell. (b) Schematic illustration of the vapor fed cell used for ORR pre-electrolysis and CO_2R, containing a gas diffusion layer (GDL) coated with a thin catalyst (Cu or Ag) as the working electrode, an anion exchange membrane (AEM), a Pt mesh as the counter electrode, and an anolyte.

Pre-electrolysis and bulk electrolysis current densities

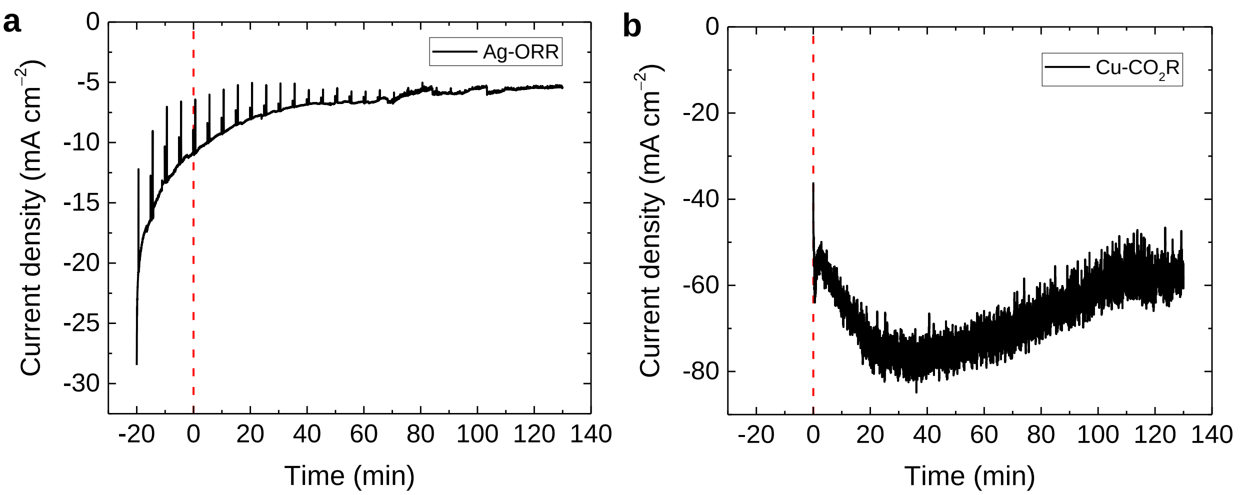

Supplementary Fig. 18 | (a) ORR pre-electrolysis current density of Ag catalyst–based gas diffusion electrode (GDE) in a vapor fed cell at an applied potential of −0.6 V vs. RHE, measured as a function of time. The initially high current density indicates the high O_2 concentration in the gas feed, which decreased over time as the remaining air in the gas stream line was evacuated by the vacuum pump. (b) CO_2R bulk electrolysis current density of Cu-based gas diffusion electrode in a vapor fed cell at an applied potential of −1.14 V vs. RHE as a function of time. The vertical red dash line in panel (a) and (b) represents the time at which the CO_2R bulk electrolysis started.

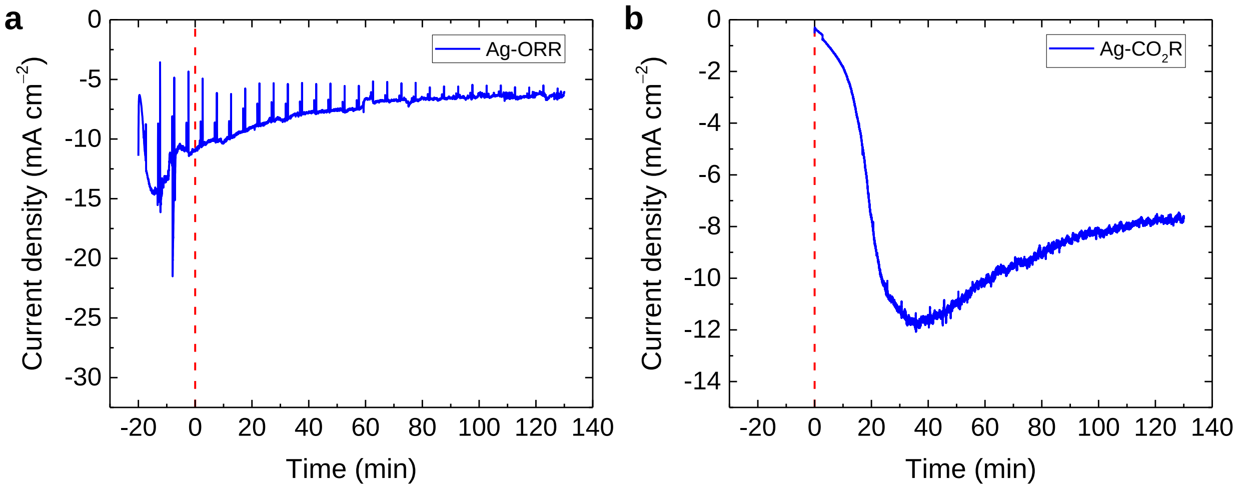

Supplementary Fig. 19 | (a) ORR pre-electrolysis current density of Ag catalyst–based gas diffusion electrode (GDE) in a vapor fed cell at an applied potential of −0.6 V vs. RHE, measured as a function of time. (b) CO_2R bulk electrolysis current density of Ag-based gas diffusion electrode in a vapor fed cell at an applied potential of −0.6 V vs. RHE as a function of time. The initially low current densities were attributed to the equilibration period for the anion exchange membrane (AEM) to fully wet which took approximately 20 minutes. The vertical red dash line indicates the time at which the CO_2R bulk electrolysis started.

Faradaic efficiency

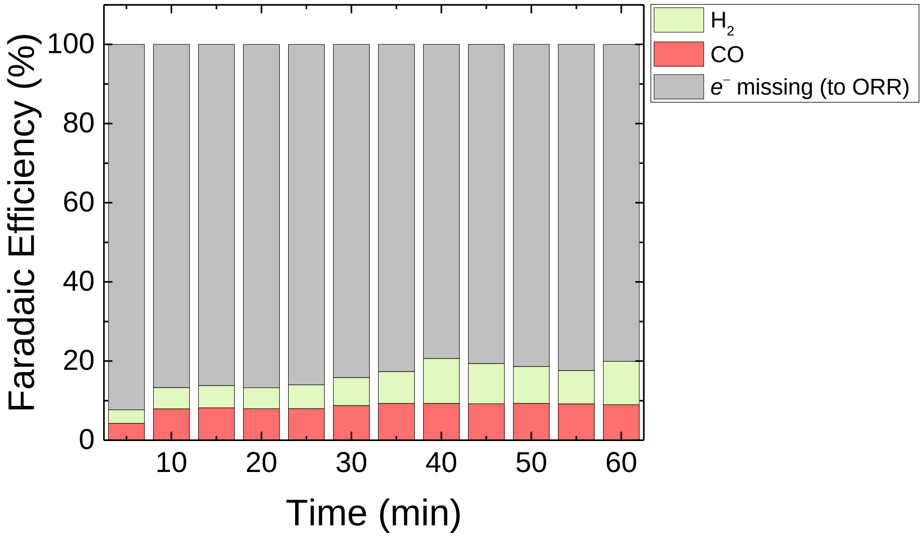

Supplementary Fig. 20 | Faradic efficiency of the Ag-catalyzed gas diffusion electrode in a vapor-fed-cell at −0.6 V vs. RHE with flowing captured gas feed directly from the BPMED (mixture composition of CO_2 93%, O_2 1.5%, N_2 6.5%). More than 80% of electrons were missing due to the preferential O_2 reduction reaction on the Ag catalyst. During the measurement, the current density varied from 14.25 to 15.35 mA cm^{-2}.

Linear sweep voltammetry of pre-electrolysis electrode

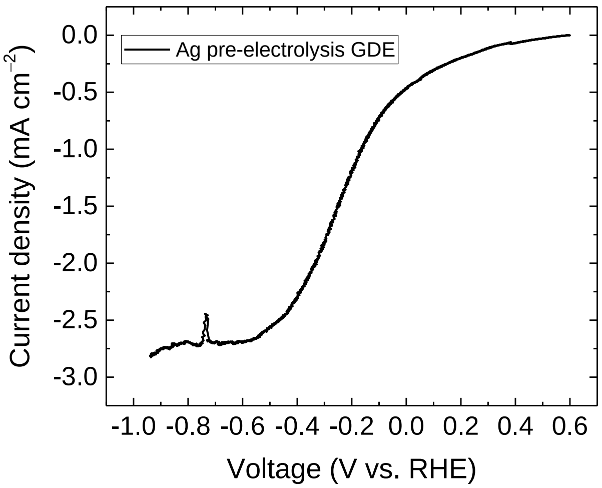

Supplementary Fig. 21 | Linear sweep voltammetry of Ag-based ORR pre-electrolysis gas diffusion electrode, showing a mass transported limit region for ORR between −0.6 and −0.8 V vs. RHE.

Supplementary Note 6

In order for the capture and conversion systems to be operationally matched, the rate of the CO_2 captured by the BPMED (G_{BPMED}) should be equal to the rate of CO_2 reduction in the electrolysis unit ($K_{\mathrm{CO_2R}}$), as given by:

$$\frac{G_{\mathrm{BPMED}}}{K_{\mathrm{CO_2R}}} = \frac{\frac{j_{\mathrm{BPMED}}A}{n_{\mathrm{BPMED}}F}\eta_{\mathrm{MC}}\frac{[\mathrm{CO_2}]}{[\mathrm{H^+}]}}{\frac{j_{\mathrm{CO_2R}}A}{n_{\mathrm{CO_2R}}F}U_{\mathrm{CO_2R}}FE} = 1 \tag{35}$$

where j_{BPMED} is the electrochemical current density of the electrodialysis unit, A is the active area (equal for the electrodialysis and electrolysis unit), n_{BPMED} is the number of participating electron to dissociate water in the BPMED ($n_{\mathrm{BPMED}} = 1$), F is the Faraday constant, η_{MC} is the membrane contactor efficiency (assumed to be 80%), $[CO_2]/[H^+]$ is the ratio between the CO_2 produced and the proton introduced in the acidification process of the BPMED (0.85 at pH 4 of natural oceanwater at DIC of 2.2 mM), $j_{\mathrm{CO_2R}}$ is the CO_2 reduction reaction current density, $n_{\mathrm{CO_2R}}$ is the number of participating electron for CO_2 reduction ($n_{\mathrm{CO_2R}} = 6$ for CO_2 reduction to ethylene or methanol) $U_{\mathrm{CO_2R}}$ is the CO_2 utilization in the electroreduction cell (assumed to be 100%), and FE is the Faradaic Efficiency (assumed to be 100%). The current density ratio factor for CO_2 reduction electrolysis relative to electrodialysis is therefore $j_{\mathrm{CO_2R}} = 4.08\, j_{\mathrm{BPMED}}$.

Supplementary Note 7

The energy penalty for a stand-alone ocean capture includes the pumping energy for oceanwater intake from the ocean, pumping energy for oceanwater filtration, pumping energy for electrodialysis process, vacuum pump energy to separate CO_2 the acidified stream and the cooling energy to condense the captured gas.

Oceanwater intake

A large-scale commercial system of indirect ocean capture can be co-located with a water desalination plant where the energy penalty for oceanwater intake, pre-treatment and pumping is paid by the desalination plant, leading to a great reduction of the capital cost, as described by Eisaman et. al.[9] In the case of a stand-alone plant where this penalty must be included, the oceanwater intake energy intensity (E_{IN}, in kWh kg^{-1} CO_2) for water desalination can be used to the determine the energy requirement to pump water from the place of intake to the plant location, as follows:

$$E_{\mathrm{IN}} = Q_{\mathrm{OW}} \frac{\rho g h}{(3.6 \times 10^{6})\eta_{\mathrm{p}}\eta_{\mathrm{m}}\dot{m}_{\mathrm{CO_2}}} \tag{36}$$

where Q_{OW} is the oceanwater flow rate (in m^3 h^{-1}), ρ is the oceanwater density (1025 kg m^{-3}), g is the acceleration of gravity (9.81 m s^{-2}), h is the total dynamic head loss (TDH, typically between 30 m and 100 m for a 1500 m long pipe.[10] A TDH value of 50 m is used in this calculation), η_{p} is the pump efficiency (94%), η_{m} is the motor efficiency (82%), and $\dot{m}_{\mathrm{CO_2}}$ is the mass flow rate of CO_2 captured (in kg h^{-1}). Assuming 78.91% capture efficiency, $Q_{\mathrm{OW}}/\dot{m}_{\mathrm{CO_2}}$ = 13.1 m^3 kg^{-1} for natural oceanwater with a DIC concentration of 2.2 mM. Using this equation, the energy consumption for oceanwater intake (E_{IN}) was calculated to be 2.37 kWh kg^{-1} CO_2.

Pre-treatment

The oceanwater received from the intake is sent through multimedia filter (including disc filter and cartridge filter), followed by ultrafiltration. During these two steps, algae, organic particles, sand particles, smaller impurities and other particles are removed.[11]

The pre-treatment energy (E_{PT}, in kWh kg^{-1} CO_2) can be estimated by determining the pump energy required to flow the oceanwater through these multistep filtration processes, as given by the following relationship:

$$E_{\mathrm{PT}} = Q_{\mathrm{OW}} \frac{(\Delta P_{\mathrm{CF}} + \Delta P_{\mathrm{DF}} + \Delta P_{\mathrm{UF}})}{36\eta_{\mathrm{p}}\eta_{\mathrm{m}}\dot{m}_{\mathrm{CO_2}}} \tag{37}$$

where ΔP_{CF} is the pressure drop in the cartridge filter (0.3 bar)[11], ΔP_{DF} is the pressure drop in the disc filter (0.22 bar)[11] and ΔP_{UF} is the pressure drop during ultrafiltration (3 bar).[11] Using this equation, the calculated energy for oceanwater pre-treatment (E_{PT}) was found to be 1.66 kWh kg^{-1} CO_2.

Centrifugal pump

Centrifugal pumps are used to flow the pre-treated oceanwater to the acidification and degasification unit. The energy consumption of the centrifugal pump (E_{P}, in kWh kg^{-1}) is equal to the energy required to

overcome the pressure drop in the BPM electrodialyzer (ΔP_{BPMED}) and the pressure drop in the membrane contactor (ΔP_{MC}), as follows:

$$E_P = Q_{OW} \frac{(\Delta P_{BPMED} + \Delta P_{MC})}{36 \eta_p \eta_m \dot{m}_{CO_2}} \tag{38}$$

Here ΔP_{BPMED} was assumed to be 0.5 bar (at maximum operating flow rate) and ΔP_{MC} was assumed to be 0.55 bar (at 70% maximum flow rate). The energy required for centrifugal pump was calculated to be 0.495 kWh kg^{-1} CO_2.

Vacuum pump

The energy consumption for the CO_2 degasification vacuum pump ($E_{VP,\,CO_2}$, in kWh kg^{-1} CO_2) is expressed as follows:[12]

$$E_{VP,\,CO_2} = \frac{F_{m,CO_2} R T \kappa_{CO_2}}{(3.6 \times 10^6)(1 - \kappa_{CO_2}) \eta_{VP} \eta_m \dot{m}_{CO_2}} \left[\left(\frac{P_{VP,\,out}}{P_{VP,\,in}} \right)^{(\kappa_{CO_2} - 1/\kappa_{CO_2})} - 1 \right] \tag{39}$$

where F_{m,CO_2} is the molar flow rate of CO_2 saturated with water vapor (in mol h^{-1}), R is the gas constant (8.314 J K^{-1} mol^{-1}), T is the temperature (in K) and κ_{CO_2} is the adiabatic constant of CO_2 (1.3). The $P_{VP,\,in}$ and $P_{VP,\,out}$ are the permeate side pressure (assumed to be 0.08 atm) and the atmospheric pressure, respectively. The η_m is the motor efficiency (82%), and $\dot{m}_{CO_2}$ is the mass flow rate of CO_2 captured (in kg h^{-1}). The efficiency of the vacuum pump (η_{VP}) is given by:[12]

$$\eta_{VP} = 0.1058 \ln \left(\frac{P_{VP,\,out}}{P_{VP,\,in}} \right) + 0.8746 = 60.74\% \tag{40}$$

The $E_{VP,\,CO_2}$ was calculated to be 0.059 kWh kg^{-1} CO_2.

The energy consumption for the stripping N_2 and O_2 from the fresh oceanwater was calculated using the assumption that G_{N_2/O_2} is 25% of G_{CO_2}, in accordance with the experimental data. The κ_{N_2/O_2} is 1.4, as given by:

$$\kappa_{N_2/O_2} = \kappa_{N_2} y_{N_2} + \kappa_{O_2} y_{O_2} \tag{41}$$

where κ_{N_2} is 1.41 and κ_{O_2} is 1.4. The mole fraction of N_2 (y_{N_2}) and O_2 (y_{O_2}) in oceanwater are assumed to be 65% and 35%. Using the same equation (Supplementary Eq. (39)), the energy consumption for N_2 and O_2 pre-degasification ($E_{VP,\,N_2/O_2}$) was found to be 0.014 kWh kg^{-1} CO_2.

Cooling

The gas is cooled upstream the vacuum pump using a vacuum pump cooling system. The energy required for cooling can be calculated as a function of the vacuum pump efficiency, as is given by:[12]

$$E_{\text{cooling}} = 0.054\eta_{\text{VP}}E_{\text{VP, CO}_2} \quad (42)$$

The calculated E_{cooling} was 0.002 kWh kg^{-1} CO_2.

Total energy penalty

The energy penalty for the balance of system of a CO_2 capture system from oceanwater in a stand-alone plant equals to the sum of the energy consumption of the individual components, as given by:

$$E_{\text{penalty, stand-alone}} = E_{\text{IN}} + E_{\text{PT}} + E_{\text{P}} + E_{\text{VP, CO}_2} + E_{\text{VP, N}_2/\text{O}_2} + E_{\text{cooling}} = 4.6 \text{ kWh kg}^{-1} \text{ CO}_2 \quad (43)$$

For a CO_2 capture system that is co-located with a water desalination plant, the energy cost for oceanwater intake, pre-treatment and pumping is assumed to be paid by the desalination plant. Thus, the energy for a co-located plant is given by:

$$E_{\text{penalty, co-located}} = E_{\text{VP, CO}_2} + +E_{\text{VP, N}_2/\text{O}_2} + E_{\text{cooling}} = 0.075 \text{ kWh kg}^{-1} \text{ CO}_2 \quad (44)$$

Supplementary Table 5 | List of energy penalty for an indirect ocean capture system that is co-located with a water desalination plant and for a stand-alone system.

Energy description	Co-located system penalty (kWh kg^{-1} CO_2)	Stand-alone system penalty (kWh kg^{-1} CO_2)
Oceanwater intake		2.370
Pre-treatment		1.660
Pumping		0.495
Vacuum pump CO_2 stripping	0.059	0.059
Vacuum pump pre-degasification O_2/N_2	0.014	0.014
Cooling	0.002	0.002
Total	0.075	4.6

Electrochemical energy

The electrochemical energy consumption for CO_2 capture from oceanwater system is given by:

$$E_{\text{electrochemical}} = \frac{VI}{(3.6 \times 10^6)\frac{I}{nF}\eta_{\text{MC}}\frac{[\text{CO}_2]}{[\text{H}^+]}\ M_{\text{CO}_2}} \quad (45)$$

where V is the voltage (V), I is the current (A), n is the number of participating electron (n = 1), F is the Faraday constant (96485.3329 A s mol^{-1}), η_{MC} is the membrane contactor efficiency (assumed to be 80%), $[CO_2]/[H^+]$ is the ratio between CO_2 produced and H^+ added in the acid compartment of the BPMED (0.85 at pH 4 for natural oceanwater with a DIC concentration of 2.2 mM) and M_{CO_2} is the molar mass of CO_2 (0.044 kg mol^{-1}). Commercial systems should operate at pH 4 to compensate for the high cost of membrane contactors and oceanwater intake, pre-treatment and pumping (Supplementary Note 8). At an industrial-scale operating current density of 200 mA cm^{-1}, the voltage was estimated to be 1.316 V (Supplementary Fig. 15), and the electrochemical energy consumption was calculated to be 1.22 kWh kg^{-1} CO_2.

Supplementary Note 8

A technoeconomic analysis of ocean capture was carried out to evaluate the viability of the proof-of-concept system using cost information that are available in the literatures. The scale of the plant was set to 880 kg-CO_2 h^{-1} or equivalent to 20 kmol-CO_2 h^{-1}, in accordance to the readily available equipment costs for CO_2 capture from reverse osmosis concentrate reported previously by Eisaman et. al.[9] The equipment size, quantity and CO_2 capture component were adjusted to an industrial scale that captures CO_2 at a rate of 880 kg h^{-1} from natural oceanwater with a DIC concentration of 2.2 mM at 78.91% capture efficiency (or membrane contactor efficiency of 80% at acidified stream pH 4.), which corresponds to an oceanwater flow rate of 276,498 m^3 day^{-1}. Note that the cost analysis presented herein only considers the cost of electrodialysis and gas stripping and ignores the cost for oceanwater pre-treatment to prevent scaling. Softening the entire oceanwater volume using ion exchanger units would be cost prohibitive at the present scale. Alternatively, feeding the basified chamber with higher flow rate of oceanwater in relative to the acidified chamber and controlling the pH of the basified chamber so that a critical pH would never be reached would minimize the divalent cation scaling in the system. Other membrane related anti-scaling materials and processes would also play an important role in the future deployment of this technology.

Supplementary Table 6 | Major equipment list for CO_2 capture from oceanwater, adjusted from a similar system previously reported by Eisaman et. al.[9] The maximum oceanwater input flow for each membrane contactor is 80 m^3 h^{-1}.

Equipment description	Qty	Inflation-adjusted cost ($ per unit)	Cost factor (-)	Purchase cost ($)
Membrane contactor	290	$7,196	2.9	$6,051,701
Electrodialyzer	4	$438,915	1.4	$2,457,925
Water condenser	2	$21,806	3.3	$143,917
Vacuum pump	2	$328,434	3.7	$2,430,409
Vacuum pump chiller	2	$124,603	2.3	$573,175

Ocean capture co-located with water desalination plant

The CO_2 capture equipment purchase cost summarized in Supplementary Table 6 reflects the direct cost. The indirect cost was assumed to be 13% of the equipment cost. Engineering, procurement and construction (EPC) was the sum of direct and indirect cost and was assumed to occur over one-year construction period. Contingency and owner's costs were assumed to be 25% and 5% of the EPC, respectively. The sum of EPC, contingency and owner's cost was equal to the total overnight cost (TOC) or the capital expenditure (CapEx). The operation and maintenance (O&M) cost was assumed to be 3.12% of CapEx per year. Major replacements include membrane contactors every 5th year, electrodialyzer stacks every 5th year, and vacuum pumps every 10th year. The electrodialyzer stack replacement cost was assumed to be 1.5 membrane costs for a system that operates at 200 mA cm^{-2} (Supplementary Eq. (33)). The electrochemical energy consumption at this current density was calculated to be 1.22 kWh kg^{-1} (Supplementary Eq. (45)) and the electricity price range was assumed to be between $0.03 and $0.06 kWh^{-1}. The plant was assumed to have a capacity factor of 90%. For an ocean capture system that is co-located with a water desalination plant, the energy costs for oceanwater intake, pre-treatment and pumping were assumed to be paid by the desalination plant, and the energy penalty for such a system only includes the energy consumption for the vacuum pump and cooling which was calculated to be 0.075 kWh kg^{-1} (Supplementary Eq. (44)). The labor cost was assumed to be equal to the previously reported full-time equivalents (FTE) to operate a 20 kmol-CO_2/hr plant, with a total of $210,000 $year^{-1}$.[9] Property taxes and insurance was assumed to be 2% of the TOC/year.

The operating costs or operating expenditure (OpEx) and product revenues depend on the levelized cost of CO_2 and were discounted to the year of construction at a discount rate of 6.5%.

$$\text{Operating Cost PV} = \sum_{i=1:n}^{n} \frac{\text{OC}_i}{(1+r)^i} \tag{46}$$

$$\text{Product revenue PV} = \sum_{i=1:n}^{n} \frac{\text{PR}_i(\text{CO}_2 \text{ levelized cost})}{(1+r)^i} \tag{47}$$

where PV is the present value, OC is the operating cost, PR is the product revenue, r is the discount rate (12%), and n is the amortization period (25 years).

A pre-tax environment was assumed, and thus depreciation was not applied to any capital assets. The levelized cost of CO_2 was calculated by adjusting its value such that the net present value of the capital and operating expenses and product revenue summed to zero. The project was assumed to be amortized over 25-year lifetime.

$$\text{NPV} = 0 = \text{Product Revenue PV (CO}_2\text{ levelized cost)} - \text{Operating Cost PV} - \text{Capital Expense} \tag{48}$$

The specific costs (in $ kg^{-1}) of the operating expenses were calculated by dividing the specific operating expense component (*e.g*. O&M, labor, electricity and replacements) present value with the sum of CapEx and the present value of all operating expenses, and multiplied with the levelized cost of CO_2.

The levelized cost of CO_2 for a system that is co-located with a water desalination plant was calculated to be in the range from \$0.50 to \$0.54 kg^{-1} CO_2. Supplementary Fig. 22a and b show the cost breakdown and the CapEx cost fraction of the co-located system, assuming an electricity price of \$0.03 kWh^{-1}.

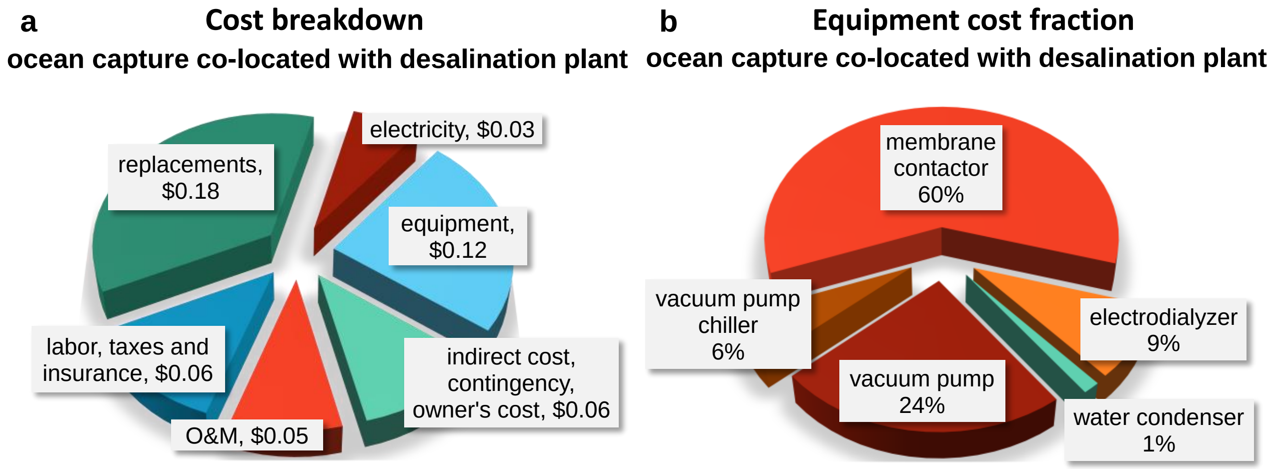

Supplementary Fig. 22 | (a) Total CO_2 cost energy breakdown and (b) Equipment cost fraction of indirect ocean capture system that is co-located with a water desalination plant, assuming an electricity price of \$0.03 kWh^{-1}.

Stand-alone ocean capture plant

In a stand-alone ocean capture, the capital and operating costs for oceanwater intake, pre-treatment and pumping were added to the previously calculated cost for a co-located plant. In principle, the processes to pump oceanwater from the place of intake to the plant location and pre-treatment units are the same as those for the water desalination, and therefore all the cost functions used for the technoeconomic analysis of a stand-alone system was assumed to be the same as those for a desalination plant. The correlation of combined oceanwater intake and pre-treatment (OWIP) capital cost (CC_{OWIP}, in 1995 USD) has been widely used:[13–17]

$$CC_{\mathrm{OWIP}} = 12659.84 {Q_{\mathrm{OW}}}^{0.8} \tag{49}$$

where Q_{OW} is the flow rate of the oceanwater feed (11,521 m^3 $h^{-1,}$ assuming a natural oceanwater DIC concentration of 2.2 mM, a CO_2 capture efficiency of 78.91%, and a plant scale of 880 kg-CO_2 h^{-1}).

Centrifugal pumps are used to feed the BPMED with the pre-treated oceanwater. The purchase cost of the centrifugal pump ($C_{\mathrm{P,\,P}}$) was estimated by the following cost function:[18]

$$C_{\mathrm{P,\,P}} = F_{\mathrm{T,\,P}} F_{\mathrm{M}} C_{\mathrm{B,\,P}} \tag{50}$$

where $F_{\mathrm{T,\,P}}$ is the pump type factor ($F_{\mathrm{T,\,P}}$ = 2 for flow rate range of 250−5000 gallons per minute (gpm), pump head range 50-500 feet (ft) and maximum motor power 250 horsepower (HP), F_{M} is the material construction factor (F_{M} = 2 for stainless steel), and $C_{\mathrm{B,\,P}}$ is the pump base cost, as given by:

$$C_{\mathrm{B,P}} = \exp\{9.171 - 0.6019\,[\ln(S)] + 0.0519\,[\ln(S)]^2\} \tag{51}$$

Here S (valid for S = 400 to S = 100000) is the size factor:

$$S = Q(H)^{0.5} \tag{52}$$

where Q is the flow rate through the pump in gpm and H is the pump head in ft of fluid flowing.

A centrifugal pump is typically driven by an electric motor whose cost is added to the pump cost (Supplementary Eq. (50)). The purchase cost of electric motor ($C_{\mathrm{P,\,M}}$) is given by:

$$C_{\mathrm{P,\,M}} = F_{\mathrm{T,\,M}} C_{\mathrm{B,\,M}} \tag{53}$$

where $F_{\mathrm{T,\,M}}$ is the motor type factor ($F_{\mathrm{T,\,M}}$ = 1.4 for totally enclosed, fan-cooled, 1 to 250 HP, 3600 rpm) and $C_{\mathrm{B,\,M}}$ is the motor base cost, as given by:

$$C_{\mathrm{B,\,M}} = \exp\{5.8259 + 0.13141\,[\ln(P_{\mathrm{C}})] + 0.053255\,[\ln(P_{\mathrm{C}})]^2 + 0.028628\,[\ln(P_{\mathrm{C}})]^3 - 0.0035549\,[\ln(P_{\mathrm{C}})]^4\} \tag{54}$$

Here P_C is the motor power consumption that is determined from the theoretical horsepower of the pump (P_T), the pump efficiency (η_P), and the electric motor efficiency (η_M):

$$P_C = \frac{P_T}{\eta_P \eta_M} = \frac{P_B}{\eta_M} = \frac{QH\rho}{33000\eta_P\eta_M} \quad (55)$$

where P_B is the pump brake horsepower and ρ is the oceanwater density in pounds per gallon. The η_P is a function of the volumetric flow rate, and η_M is a function of the pump brake horsepower:

$$\eta_P = -0.316 + 0.24015\,(\ln Q) - 0.01199\,(\ln Q)^2 \quad (56)$$

for Q in the range of 50 to 5000 gpm, and:

$$\eta_M = 0.8 + 0.0319\,(\ln P_B) - 0.00182\,(\ln P_B)^2 \quad (57)$$

for P_B in the range of 1 to 1500 HP.

The centrifugal pump and electric motor cost functions above are described in 2006 dollars with an average cost index of 500 (CE = 500),[18] and were adjusted to 2019 dollars using an average cost index of 607.5 (CE = 607.5).

The centrifugal pump capital cost (CC_P) is then given by the sum of the purchase cost of pump and motor:

$$CC_P = C_{P,P} + C_{P,M} \quad (58)$$

The oceanwater waste described in this work was assumed to have almost the same mineral composition as the fresh oceanwater, except restored alkalinity, and therefore may be discharged to open water body or near-shore zone without the need for post-treatment, concentrate management, construction of lengthy outfalls and costly diffuser structures. Additionally, the oceanwater output from the BPMED was assumed to carry a residual pressure of 2 bar, and therefore no additional pumping is required for surface water discharge.

The total capital cost for oceanwater intake, pre-treatment and pumping (CC_{OWIPP}) is expressed by the sum of the these cost functions, multiplied with 1.441 to represent the indirect cost:[13,19]

$$CC_{OWIPP} = 1.441(CC_{OWIP} + CC_P) \quad (59)$$

The annual operating costs (OC) for oceanwater intake, pre-treatment and pumping include the cost of electricity ($OC_{electricity}$), insurance ($OC_{insurance}$), maintenance ($OC_{maintenance}$) and labor (OC_{labor}), as given by:[19,20]

$$OC_{electricity} = 365(E_{IN} + E_{PT} + E_P)\dot{m}_{CO_2} C_e f_c \quad (60)$$

$$OC_{\mathrm{maintenance}} = 0.01 \times 365 Q_{\mathrm{OW}} f_{\mathrm{c}} \quad (61)$$

$$OC_{\mathrm{insurance}} = 0.005\ CC_{\mathrm{OWIPP,\ total}} \quad (62)$$

$$OC_{\mathrm{labor}} = 0.01 \times 365 Q_{\mathrm{OWIP}} f_{\mathrm{c}} \quad (63)$$

where E_{IN}, E_{PT}, E_{P} are the energy for oceanwater intake, pre-treatment and pumping, respectively, as described in Supplementary Note 7 (Supplementary Eq. (36), (37) and (38)). The $\dot{m}_{CO_2}$ is the mass flow rate of the CO_2 output or the CO_2 production scale of the plant (880 kg h^{-1}), C_e is the cost of electricity (assumed to be between \$0.03 and \$0.06 kWh^{-1}) and f_c is the capacity factor (90%).

Combining all the expenses for oceanwater intake, pre-treatment, pumping and the costs for the electrodialysis and gas stripping units, and assuming a discount rate of 6.5% for a plant lifetime of 25 years with 90% capacity factor, the levelized cost of CO_2 was calculated to be \$1.87 and \$2.05 kg^{-1} CO_2 for an electricity price of \$0.03 and \$0.06 kWh^{-1}, respectively.

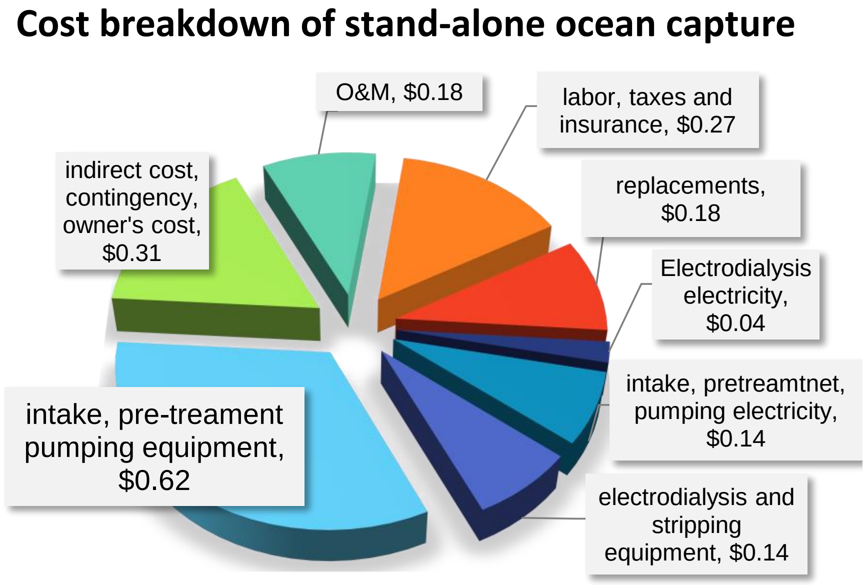

Supplementary Fig. 23 | CO_2 cost energy breakdown of a stand-alone indirect ocean capture plant, including the CapEx and OpEx for electrodialysis, gas stripping, oceanwater intake, pre-treatment and pumping, at an electricity price of \$0.03 kWh^{-1}.

Supplementary Table 7 | Summary of the cost breakdown of the co-located and stand-alone systems.

Ocean capture scenario	CapEx (\$ kg^{-1} CO_2)	OpEx (\$ kg^{-1} CO_2)				Levelized cost CO_2 (\$ kg^{-1})
		O & M	Labor, tax, insurance	Replacements	Electricity	
Co-located	0.18	0.05	0.06	0.18	0.04[a]-0.08[b]	0.50[a] -0.54[b]
Stand-alone	1.07	0.18	0.27	0.18	0.18[a] -0.35[b]	1.87[a]-2.05[b]

[a] Electricity price of \$0.03 kWh^{-1}.

[b] Electricity price of \$0.06 kWh^{-1}.

Supplementary Note 9

A recent analysis by Eisaman suggests that a stand-alone land-based ocean capture (OC) is less energetically and economically advantageous than direct air capture (DAC).[10] This is primarily due to the higher density of oceanwater compared to air, meaning that more energy is required to pump oceanwater than to pump air. For example, the energy required for CO_2 capture process (E) can be simplified into the following equation:

$$E = E_\mathrm{P} + E_\mathrm{Z} = \frac{Q\rho g h}{\dot{m}_{\mathrm{CO_2}}\eta} + E_\mathrm{Z} \tag{64}$$

where E_P is the pumping energy and E_Z process specific energy. Q is the volumetric flow rate (in $\mathrm{m^3\ h^{-1}}$) of the CO_2 source medium (air or oceanwater), ρ is the density of oceanwater or air ($\rho_\mathrm{oceanwater} = c = 1025$ kg m^{-3} and $\rho_\mathrm{air} = \rho^\mathrm{DAC} = 1.225$ kg m^{-3}), g is the acceleration of gravity (9.81 m s^{-2}), h is the pump head loss (h^OC = 50 m for intake + 35 m for pre-treatment + 10.5 m for feed pumping = 95.5, m and h^DAC = 67. Pa/1.2 kg m^{-3} × 9.81 m s^{-2} = 5.8 m), $\dot{m}_{\mathrm{CO_2}}$ is the mass flow rate of the CO_2 captured (in kg h^{-1}), η is the pumping efficiency (assumed to be equal for OC and DAC, $\eta^\mathrm{OC} = \eta^\mathrm{DAC}$) and E_Z is the electrochemical energy for OC ($E_\mathrm{Z}{}^\mathrm{OC}$ = 1.25 kWh kg^{-1} CO_2 at 200 mA cm^{-2}), and regeneration energy for DAC ($E_\mathrm{Z}{}^\mathrm{DAC}$ = 5.25 GJ t^{-1} CO_2 = 1.46 kWh kg^{-1} CO_2).[21] Note that Supplementary Eq. (64) only considers the most important energy and does not take into account the non-negligible energy needed in the process. Supplementary Eq. (64) can be modified as follows:

$$E = \frac{\rho g h}{D\eta} + E_Z \tag{65}$$

where D is the mass density of CO_2 in oceanwater and in air (D^OC = 0.0968 kg-CO_2 m^{-3} of oceanwater and D^DAC 0.000733 kg-CO_2 m^{-3} of air). The individual parameter factors of OC relative to DAC are given by: $\rho^\mathrm{OC} = 836.7\rho^\mathrm{DAC}$, $h^\mathrm{OC} = 16.5\ h^\mathrm{DAC}$, $D^\mathrm{OC} = 132D^\mathrm{DAC}$, $\eta^\mathrm{OC} = \eta^\mathrm{DAC}$ and $E_\mathrm{Z}{}^\mathrm{OC} = 0.86\ E_\mathrm{Z}{}^\mathrm{DAC}$.

Therefore, the energy consumption for OC relative to DAC can be expressed by:

$$E^\mathrm{OC} = 104.6E_\mathrm{P}{}^\mathrm{DAC} + 0.86E_\mathrm{Z}{}^\mathrm{DAC} \tag{66}$$

Supplementary Eq. (66) indicates that the energy penalty for OC mostly arises from the energy consumption for pumping oceanwater. This is primarily due to the higher density of oceanwater relative to air (ρ^OC = $836.7\rho^\mathrm{DAC}$) outweighs the mass concentration of CO_2 in oceanwater ($D^\mathrm{OC} = 132D^\mathrm{DAC}$), and thus more energy required to capture CO_2 in oceanwater than in air.

That said, ocean capture can be energetically competitive against direct air capture if the ocean capture system is co-located with a water desalination plant where the energy for oceanwater intake, pre-treatment and feed pumping is paid by the desalination plant, resulting in $E^\mathrm{OC} = 0.86E_\mathrm{Z}{}^\mathrm{DAC} - E_\mathrm{P}{}^\mathrm{DAC}$.

Supplementary Note 10

The thermodynamic potential limit to acidify oceanwater to a mild pH of 4.6 is given by $V_{\text{limit, direct}}$ = 0.059 (pH 10.6 − pH 4.6) = 0.354 V, where pH 10.6 represents the restored alkalinity pH of the basified stream, assuming maximum CO_2 removal from the acidified stream, and pH 4.6 represents the acidified stream pH at which the ratio between [CO_2] produced and [H^+] added ([CO_2]/[H^+]) in the acid compartment of the BPMED is the highest (Supplementary Note 5).

The thermodynamic energy requirement for a direct CO_2 capture from oceanwater by BPMED is then given by:

$$E_{\text{limit, direct}} = \frac{V_{\text{limit, direct}}\, I\ (\text{kW})}{G_{\text{CO}_2}\left(\frac{\text{kg}}{\text{h}}\right)} = \frac{0.354\ (\text{V})\ I\ (\text{A})\ 10^{-3}\left(\frac{\text{kW}}{\text{W}}\right)}{\frac{I\ (\text{A})}{nF\left(\frac{\text{A s}}{\text{mol}}\right)}\frac{[\text{CO}_2]}{[\text{H}^+]}3600\ \left(\frac{\text{s}}{\text{hour}}\right)M_{\text{CO}_2}\left(\frac{\text{kg}}{\text{mol}}\right)} = 0.25\frac{\text{kWh}}{\text{kg CO}_2} \tag{67}$$

where V is the voltage, I is the current, n is the number of participating electron (n = 1), F is the Faraday constant (96485.3329 A s mol^{-1}), [CO_2]/[H^+] is the ratio between CO_2 produced and H^+ added in the acid compartment (0.87 at pH 4.6 for natural oceanwater with a DIC concentration of 2.2 mM) and M_{CO_2} is the molar mass of CO_2 (0.044 kg mol^{-1}).

For indirect process, where 1 M HCl and 1 M NaOH are separately generated to acidify and basify oceanwater using BPMED, the thermodynamic potential limit is given by $V_{\text{limit, indirect}}$ = 0.059 (pH 14 − pH 0) = 0.826 V. Hence the energy requirement for the indirect acidification process BPMED is:

$$E_{\text{limit, indirect}} = \frac{V_{\text{limit, indirect}}\, I\ (\text{kW})}{G_{\text{CO}_2}\left(\frac{\text{kg}}{\text{h}}\right)} = \frac{0.826\ (\text{V})\ I\ (\text{A})\ 10^{-3}\left(\frac{\text{kW}}{\text{W}}\right)}{\frac{I\ (\text{A})}{nF\left(\frac{\text{A s}}{\text{mol}}\right)}\frac{[\text{CO}_2]}{[\text{H}^+]}3600\ \left(\frac{\text{s}}{\text{hour}}\right)M_{\text{CO}_2}\left(\frac{\text{kg}}{\text{mol}}\right)} = 0.6\frac{\text{kWh}}{\text{kg CO}_2} \tag{68}$$

Supplementary References

1. Willauer, H. D., DiMascio, F., Hardy, D. R. & Williams, F. W. Development of an Electrolytic Cation Exchange Module for the Simultaneous Extraction of Carbon Dioxide and Hydrogen Gas from Natural Seawater. *Energy & Fuels* **31**, 1723–1730 (2017).

2. Eisaman, M. D. *et al.* CO_2 extraction from seawater using bipolar membrane electrodialysis. *Energy Environ. Sci.* **5**, 7346 (2012).

3. Craig, N. Electrochemical Behavior of Bipolar Membranes. *UC Berkeley Electronic Theses and Dissertations* (2013).

4. DOE (1994). *Handbook of methods for the analysis of the various parameters of the carbon dioxide system in sea water; version 2, A. G. Dickson & C. Goyet, eds., ORNL/CDIAC-74.*

5. Zeebe, R. E. & Wolf-Gladrow, D. CO_2 in seawater: Equilibrium, Kinetics, Isotopes. in *Elsevier Oceanography Series, 65* (2001).

6. Pilson, M. E. Q. *An Introduction to the Chemistry of the Sea*. *An Introduction to the Chemistry of the Sea* (Cambridge University Press, 2012).

7. Liberti, A. & Light, T. S. Potentiometric determination of *K*w with the glass electrode. *J. Chem. Educ.* **39**, 236 (1962).

8. Tanaka, Y. *Ion Exchange Membranes*. (Elsevier, 2015).

9. Eisaman, M. D. *et al.* Indirect ocean capture of atmospheric CO_2: Part II. Understanding the cost of negative emissions. *Int. J. Greenh. Gas Control* **70**, 254–261 (2018).

10. Eisaman, M. D. Negative Emissions Technologies: The Tradeoffs of Air-Capture Economics. *Joule* **4**, 516–520 (2020).

11. Shahabi, M. P., McHugh, A. & Ho, G. Environmental and economic assessment of beach well intake versus open intake for seawater reverse osmosis desalination. *Desalination* **357**, 259–266 (2015).

12. Matsumiya, N., Teramoto, M., Kitada, S. & Matsuyama, H. Evaluation of energy consumption for separation of CO_2 in flue gas by hollow fiber facilitated transport membrane module with permeation of amine solution. *Sep. Purif. Technol.* **46**, 26–32 (2005).

13. Malek, A., Hawlader, M. N. A. & Ho, J. C. Design and economics of RO seawater desalination. *Desalination* **105**, 245–261 (1996).

14. Marcovecchio, M. G., Aguirre, P. A. & Scenna, N. J. Global optimal design of reverse osmosis networks for seawater desalination: Modeling and algorithm. *Desalination* (2005).

15. Vince, F., Marechal, F., Aoustin, E. & Bréant, P. Multi-objective optimization of RO desalination plants. *Desalination* (2008).

16. Kim, Y. M. *et al.* Development of a package model for process simulation and cost estimation of seawater reverse osmosis desalination plant. *Desalination* (2009).

17. Wan, C. F. & Chung, T.-S. Techno-economic evaluation of various RO+PRO and RO+FO integrated processes. *Appl. Energy* **212**, 1038–1050 (2018).

18. Seider, W. D., Seader, J. D., Lewin, D. R. & Widagdo, S. *Product and Process Design Principles - Synthesis, Analysis and Evaluation*. (John Wiley & Sons, Ltd, 2009).

19. Kim, J. & Hong, S. A novel single-pass reverse osmosis configuration for high-purity water production and low energy consumption in seawater desalination. *Desalination* **429**, 142–154 (2018).

20. Du, Y., Xie, L., Liu, Y., Zhang, S. & Xu, Y. Optimization of reverse osmosis networks with split partial second pass design. *Desalination* (2015).

21. Keith, D. W., Holmes, G., St. Angelo, D. & Heidel, K. A Process for Capturing CO_2 from the Atmosphere. *Joule* **2**, 1573–1594 (2018).
